# GATA3-dependent cellular reprogramming requires activation-domain dependent recruitment of a chromatin remodeler

**DOI:** 10.1186/s13059-016-0897-0

**Published:** 2016-02-27

**Authors:** Motoki Takaku, Sara A. Grimm, Takashi Shimbo, Lalith Perera, Roberta Menafra, Hendrik G. Stunnenberg, Trevor K. Archer, Shinichi Machida, Hitoshi Kurumizaka, Paul A. Wade

**Affiliations:** Epigenetics and Stem Cell Biology Laboratory, National Institute of Environmental Health Sciences, Research Triangle Park, NC USA; Integrative Bioinformatics, National Institute of Environmental Health Sciences, Research Triangle Park, NC USA; Laboratory of Genome Integrity and Structural Biology, National Institute of Environmental Health Sciences, Research Triangle Park, NC USA; Department of Molecular Biology, Faculties of Science and Medicine, Radboud University, Nijmegen, Netherlands; Laboratory of Structural Biology, Graduate School of Advanced Science and Engineering, Waseda University, Tokyo, Japan

**Keywords:** GATA3, Mesenchymal-to-epithelial transition, Breast cancer, Pioneer factor, Chromatin remodeling, Enhancer establishment

## Abstract

**Background:**

Transcription factor-dependent cellular reprogramming is integral to normal development and is central to production of induced pluripotent stem cells. This process typically requires pioneer transcription factors (TFs) to induce *de novo* formation of enhancers at previously closed chromatin. Mechanistic information on this process is currently sparse.

**Results:**

Here we explore the mechanistic basis by which GATA3 functions as a pioneer TF in a cellular reprogramming event relevant to breast cancer, the mesenchymal to epithelial transition (MET). In some instances, GATA3 binds previously inaccessible chromatin, characterized by stable, positioned nucleosomes where it induces nucleosome eviction, alters local histone modifications, and remodels local chromatin architecture. At other loci, GATA3 binding induces nucleosome sliding without concomitant generation of accessible chromatin. Deletion of the transactivation domain retains the chromatin binding ability of GATA3 but cripples chromatin reprogramming ability, resulting in failure to induce MET.

**Conclusions:**

These data provide mechanistic insights into GATA3-mediated chromatin reprogramming during MET, and suggest unexpected complexity to TF pioneering. Successful reprogramming requires stable binding to a nucleosomal site; activation domain-dependent recruitment of co-factors including BRG1, the ATPase subunit of the SWI/SNF chromatin remodeling complex; and appropriate genomic context. The resulting model provides a new conceptual framework for *de novo* enhancer establishment by a pioneer TF.

**Electronic supplementary material:**

The online version of this article (doi:10.1186/s13059-016-0897-0) contains supplementary material, which is available to authorized users.

## Background

Transcription factors (TFs) are utilized throughout life as a means of regulating transcriptional output. The human genome encodes somewhere between 1,500 and 2,000 TFs [1], making them one of the more abundant protein classes. These proteins recognize DNA motifs in and around genes and alter RNA synthesis through a variety of mechanisms [2, 3]. Classification based on structures of their DNA-binding domains provides cardinal insights into their modes of DNA recognition. While structural studies have advanced our understanding of how TFs interact with DNA, genomic studies of their localization indicate that the vast majority of potential TF binding sites are not occupied *in vivo* [4–6].

In eukaryotes, genomic DNA is packaged into nucleosomes, the fundamental unit of chromatin structure. Nucleosomal architecture presents a barrier to successful protein-DNA interactions for many TFs [7–9]. Pioneer TFs represent an exception to this general rule; these proteins have the ability to interact productively with DNA in a nucleosomal context. Pioneer factors have at least three fundamental properties as recently described by Iwafuchi-Doi and Zaret [10]: (1) they can interact with their cognate recognition sequences in nuclease-resistant chromatin prior to activation of transcription; (2) they increase local chromatin accessibility as a prelude to productive binding of other factors; and (3) they have integral roles in the establishment of cell lineage. FOXA1 represents a prototypical example of a pioneer TF; FOXA1 can bind nucleosomal DNA *in vitro*, and target closed chromatin regions *in vivo* which subsequently become accessible and permissive for productive binding of other factors [5, 11–13]. FOXA1 is required for multiple developmental lineage commitment choices including those leading to development of liver, lung, and mammary gland [13–17]. Other TFs involved in reprogramming of somatic cells to pluripotency such as Oct4, Sox2, and Klf4, also function as pioneer factors utilizing unique adaptations of their DNA recognition surfaces to accommodate nucleosome structure, which likely represents a common genome interrogation mechanism of the pioneer class [18, 19]. FOXA1 has structural similarity to linker histone [20, 21], therefore it is thought to compete with linker histone [12]. However validated pioneer factors exhibit large structural diversity in sequence-recognition domains, mechanism(s) underlying chromatin opening may vary.

DNA-binding proteins that have zinc finger domains compose the most abundant protein family in eukaryotic genomes [22]. The vertebrate GATA family proteins, which possess two zinc-finger domains, play critical roles across development and differentiation, contributing to lineage commitment. They also possess several hallmark features, as a family, of pioneer TFs: several family members can bind to nucleosomal DNA and induce accessibility, both *in vivo* and *in vitro* [11, 23–29]. Importantly the DNA-binding domains of GATA family proteins are structurally distinct from FOXA1, implying that mechanism(s) to create open chromatin may differ. While elegant evidence supports an intrinsic capacity of FOXA1 to remodel chromatin without the assistance of co-factors [11], it remains unclear how and whether GATA proteins induce similar structural alterations.

In this study, we focus on the human GATA3 protein, which is the only human GATA family member whose DNA-binding domain has been crystallized [30]. GATA3 is a key regulator of multiple developmental pathways including mammary epithelial cell differentiation, T lymphocyte development, and trophoblast development [31–34]. GATA3 is implicated in breast cancer progression, and recently has been identified as one of the most frequently mutated genes in breast cancer [35]. A considerable number of clinical studies have demonstrated that reduced expression level of GATA3 correlates with poor prognosis [36]. Consistent with the *in vivo* data, GATA3 exerts metastasis suppressive functions when expressed exogenously in a human basal-like breast cancer cell line by inducing the mesenchymal-to-epithelial transition (MET) [37–39]. Although this transition is accompanied by gene expression reprogramming including microRNA induction [37], the molecular mechanisms underlying this cell reprogramming process at the chromatin level are incompletely understood. In other contexts, GATA3 is known to reprogram fibroblasts to induced pluripotent stem cells (iPSCs) [40] as well as induced trophoblast stem cells (iTSCs) [41, 42] in collaboration with other TFs, implying a potential role for GATA3 in chromatin reprogramming.

In order to investigate the impact(s) of GATA3 on chromatin, we utilized the MET model in the MDA-MB-231 breast cancer cell line. Consistent with previous results, GATA3 expression induced mesenchymal-to-epithelial transition at the cellular and molecular level and mediated reprogramming of chromatin at genes integral to MET. Our studies provide experimental evidence that GATA3 can direct alterations in local chromatin during the MET reprogramming process, including pioneer factor activity, that differ depending on local context and that these alterations proceed through an intermediate consisting of nucleosomal DNA bound by a pioneer factor.

## Results

### GATA3 binding sites acquire open chromatin structure and enhancer-like histone modifications

**Fig. 1:**
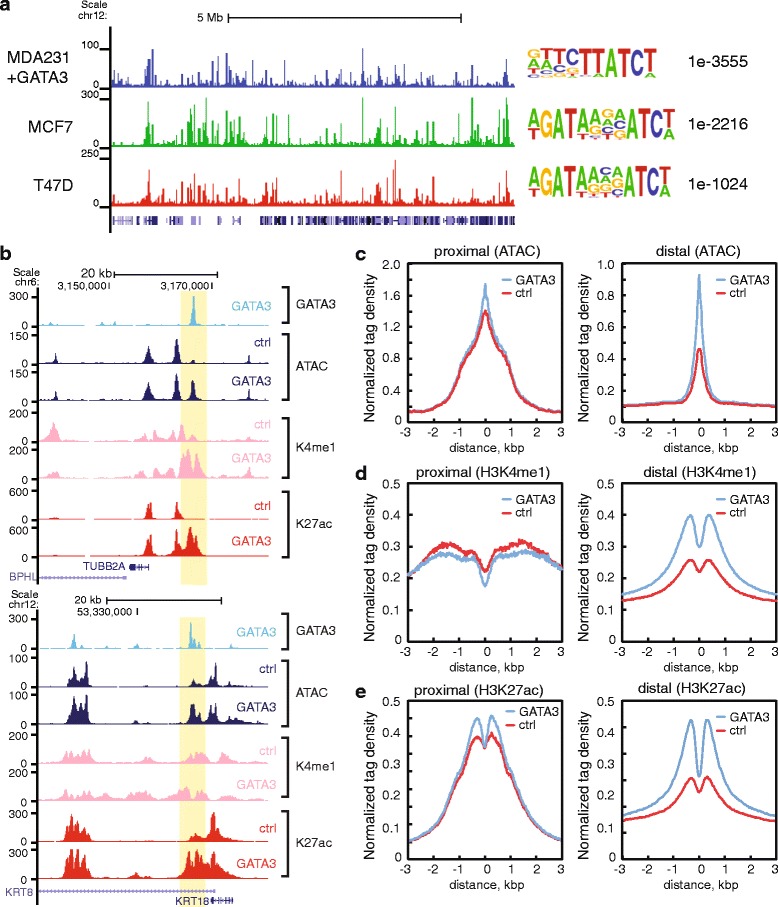
Chromatin domain bound by GATA3 becomes more accessible together with increased levels of enhancer-like histone marks. **a** Genomic localization of GATA3 across three breast cancer cell lines. UCSC Genome Browser snapshot showing the mapped read coverage of GATA3 ChIP-seq in MDA-MB-231 (blue), MCF7 (green), and T47D cells (red). The top motif identified by HOMER *de novo* motif analysis is represented at the right with *P* values. **b** Representative genomic loci acquired DNA accessibility. The GATA3-binding sites displayed: (top) significant increased level or (bottom) *de novo* peaks of ATAC-seq, H3K4me1, and H3K27ac signals after ectopic expression of GATA3. The other example loci are shown in Additional file 1: Figure S1. Metagene profiles of normalized ATAC-seq (**c**), H3K4me1 ChIP-seq (**d**), and H3K27ac ChIP-seq (**e**) are shown to compare the average signal levels centered on GATA3 ChIP-seq peaks in control and GATA3 expressing cells. The GATA3 peaks are divided into TSS-proximal (less than or equal to 1 kb) or distal (more than 1 kb) peaks based on the distance

To investigate chromatin reprogramming during the MET process, we utilized MDA-MB-231 breast cancer cells, in which GATA3, and its known partners, FOXA1 and estrogen receptor-α, are below limits of detection at the protein level. Previous work has demonstrated that ectopic expression of GATA3 promotes cell reprogramming (MET) in MDA-MB-231 cells with an alteration in metastatic capacity [37–39]. We first established stable cell lines expressing wild-type GATA3 at levels comparable to that found in GATA3-positive breast cancer cells (Additional file 1: Figure S1A). As expected, GATA3-expressing cells were reprogrammed, exhibiting phenotypic changes at the cellular and molecular level (Additional file 1: Figure S1B, S1C). In order to determine the genomic distribution of GATA3 in MDA-MB-231 cells, we performed ChIP-seq analyses, identifying 43,504 peaks of enrichment (Fig. 1a; Additional file 1: Figure S1D). GATA3 localization following exogenous expression in MDA-MB-231 cells was compared to the pattern previously observed in the luminal epithelial breast cancer cell lines T47D and MCF7 [43]. We detected a large number of cell type specific binding sites along with many binding sites that overlapped those in GATA3-positive cell lines (Fig. 1a; Additional file 1: Figure S1E). *De novo* motif analysis of the peaks in MDA-MB-231 cells revealed significant enrichment of the GATA3 consensus binding site (Fig. 1a). Therefore we concluded that GATA3 bound to chromatin in MDA-MB-231 cells, and this system likely captures critical regulatory features of cellular reprogramming from a mesenchymal state to an epithelial one.

Pioneer factors are characterized by their ability to bind to previously ‘closed’ chromatin and elicit a chromatin state change. To evaluate the impact of GATA3 on chromatin structure, we measured chromatin accessibility in MDA-MB-231 cells with and without GATA3 expression using ATAC-seq [44]. At the individual locus level, many GATA3-binding sites including MET associated gene loci exhibited increased accessibility in GATA3-expressing cells (hereafter MDA231-GATA3 cells) (Fig 1b; Additional file 1: Figure S1F). Across the genome, TSS-distal peaks (greater than 1 kb from nearest TSS) exhibited a significant difference (*P* values < e-10) between control and MDA231-GATA3 cells (Fig. 1c). Randomly selected GATA motif containing regions showed similar levels of transposase sensitivity between control and GATA3-expressing cells, indicating that overall transposase integration efficiencies were similar (Additional file 1: Figure S1G). To dissect the function of chromatin domains bound by GATA3, we investigated histone modifications typically associated with active chromatin at enhancers, such as H3K4 monomethylation (H3K4me1) and H3K27 acetylation (H3K27ac). Similar to the ATAC-seq results, H3K4me1 and H3K27ac levels in MDA-231-GATA3 cells were drastically increased at TSS distal GATA3 peaks (Fig. 1d, e).

### Chromatin structure based classification reveals a pioneer class of binding sites

**Fig. 2:**
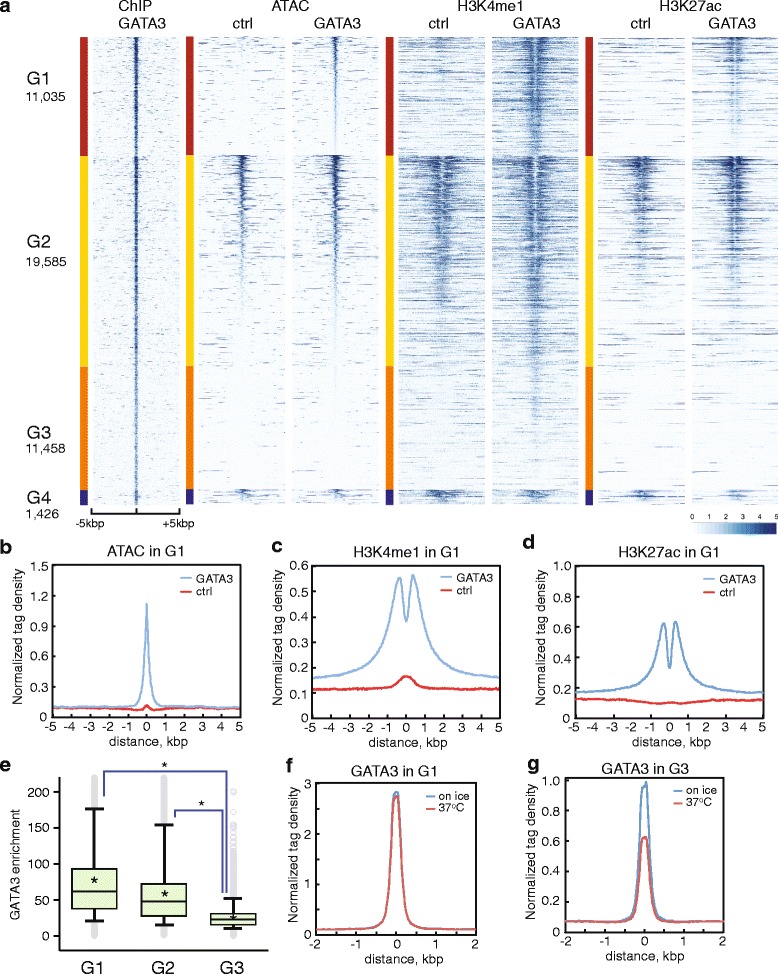
Identification and characterization of ‘Pioneer’ sites bound by GATA3. **a** Read density heatmaps showing the signal enrichment of GATA3 ChIP-seq, ATAC-seq, H3K4me1, and H3K27ac ChIP-seq. Classification from G1 to G4 was carried out based on ATAC-seq signal changes between control and GATA3 expressing cells. The number of peaks in each category is reported below the group label. Each row indicates a 10 kb window centered on a GATA3 binding site. The scale of read density after normalization (see Additional file 1: Table S6) is indicated at the bottom right. Metagene profiles of normalized ATAC-seq (**b**), H3K4me1 ChIP-seq (**c**), and H3K27ac ChIP-seq (**d**) in G1 are shown for the comparison of the average signal levels in control and GATA3 expressing cells. Metagene profiles in the other groups are shown in Additional file 1: Figure S2. **e** The signal enrichment of GATA3 ChIP-seq in each group is shown as a box-and-whisker plot. The top and bottom whiskers show 5th and 95th percentile, respectively. The horizontal line and cross mark the median and mean. *P <0.0001, Mann-Whitney test. Metagene plots of normalized tag density of GATA3 ChIP-seq in G1 (**f**) and G3 (**g**) performed with or without thermal treatment at 37 °C. The metagene plot in G2 and the quantitative analysis are represented in Additional file 1: Figure S2

To dissect the chromatin bound by GATA3, we classified the GATA3 peaks into four groups based on ATAC-seq signal changes before and after the epithelial transition. At a FDR <0.1, fold change >2, 11,035 GATA3 peaks (25 %) became sensitive to the transposase (Fig. 2a, G1). A total of 1,426 binding sites (3 %) displayed decreased levels of ATAC-seq signals following GATA3 expression (Fig. 2a, G4). The majority of binding sites (31,043 peaks, 71 %) did not change transposase sensitivity; these sites were subclassified into signal positive (19,585 loci, 45 %) and negative (11,458 loci, 26 %) groups based on the signal intensity in control cells (cutoff: read counts <20) (Fig. 2a, G2 and G3). We also interrogated histone marks in each group. Heatmap and metagene plot analyses revealed that the category with increased ATAC sensitivity, G1, had increased levels of H3K4me1 and H3K27ac following GATA3 expression (Fig. 2a-d). The signal conserved groups (G2 and G3) showed modest increases in the level of H3K4me1 and H3K27ac (Additional file 1: Figure S2D, S2E, S2G, S2H). The class of loci where accessibility decreased exhibited decreases in both H3K4me1and H3K27ac (Additional file 1: Figure S2C, S2F, S2I). These analyses demonstrate that GATA3-bound loci can be classified based on whether or not they were previously accessible and whether local chromatin was altered following introduction of GATA3. One category, G1, exhibits the hallmark properties expected for pioneering activity.

To elucidate the correlation between gene expression and GATA3 binding, we assigned GATA3 peaks to target genes using a simple distance metric, and measured the impact of GATA3 on gene expression using RNA-seq. GATA3-binding events in category G1 were enriched for increased gene expression, and the ratio of the genes categorized as upregulated was higher in G1 than that in G2 and G3 (Additional file 1: Figure S3A-D). Interestingly, target genes in G1 contain transcripts indicative of reprogramming of MDA-MB-231 to an epithelial identity, such as KRT8, KRT18, CDH1, and CLDN3 (Additional file 2: Table S1). When analyzed over time following exogenous expression of GATA3 using lentivirus, a subset of those genes were upregulated within 48-96 h after the induction of GATA3 (Additional file 1: Figure S3E). Functional pathway analysis of genes found in G1 featured enrichment in genes integral to the MET and to maintenance of epithelial identity (Additional file 1: Figure S3F). These functional categories were not enriched in G3 (Additional file 1: Figure S3G, Additional file 2: Table S1), suggesting that GATA3 binding and chromatin opening at G1 sites is integral to the MET, consistent with function as a pioneer.

### GATA3 remodels local nucleosome structure

Mechanistically, it was unclear why GATA3 binding at previously inaccessible loci, as detected by ChIP-seq, should sometimes, but not always, result in formation of accessible chromatin. Therefore, we sought to understand why GATA3 could open chromatin structure only at G1 sites but not at G3 sites. To address this question, we compared the enrichment of GATA3 between these groups. The open chromatin loci within categories G1 and G2 had similar enrichment of GATA3; the transposase-inaccessible loci bound by GATA3 in category G3 clearly displayed lower enrichment (Fig. 2e; Additional file 1: Figure S2J), suggesting a fundamental difference in the interaction of GATA3 with its cognate recognition element at these two classes of sites. These observed differences in ChIP-seq read level could result from differences in inherent affinity driven by local DNA sequence. Alternatively, they could result from interaction of GATA3 with nucleosomal DNA (G3) fundamentally differing from interaction with accessible chromatin (G1 and G2). Finally, the differences in enrichment could result from differential association with co-factors, such as the Friend of GATA proteins [45, 46] or other partners. Investigating the genes differentially expressed in our system (Additional file 1: Figure S1C), 108 known TFs were upregulated in MDA-231-GATA3 cells, and two TF motifs were co-enriched in category G1, indicating the potential for cooperative action at a subset of sites (Additional file 3: Table S2).

Qualitatively, consensus motifs derived from the loci in G1 did not differ substantially from those in G3 (Additional file 1: Table S3), suggesting differences in binding do not result from deviation of recognition elements from consensus. Accordingly, we hypothesized that the observed difference in enrichment might result from either difference in co-factor association or from difference in substrate quality when comparing nucleosomal DNA to disrupted chromatin. Either of these two possible outcomes should result in an alteration in apparent binding affinity at categories G1 and G2 when compared to category G3. To test this hypothesis, we asked whether increasing thermodynamic entropy would differentially impact category G3 as opposed to categories G1 and G2. Accordingly, we briefly incubated nuclei at 37^o^ C or on ice in physiological salt prior to cross linking and performed ChIP-seq. We observed that binding events in category G3, and only G3, were significantly diminished by increasing temperature (Fig. 2f, g, Additional file 1: Figure S2K, S2L). These data suggest that the on and off rate constants at category G3 differ from those at categories G1 and G2. This scenario is consistent with a model wherein category G3 represents GATA3 bound at its recognition site in the context of canonical nucleosomal architecture whereas categories G1 and G2 fundamentally differ in the nature of the substrate. Utilizing our previous ChIP-seq data [43] and ENCODE DNase-seq data [2], we confirmed the presence of a category of G3-like GATA3 bound sites in luminal breast cancer cells (T47D and MCF7 cells) (Additional file 1: Figure S2M, S2N). Of the GATA3 peaks, 51 % (in the case of MCF7) and 37 % (in the case of T47D) did not overlap with DNase-seq peaks in these cells, suggesting the existence of loci that are bound by GATA3 but not accessible, like category G3 in the MDA-231 reprogramming system, is unlikely to be an artefact of expression out of normal context.

**Fig. 3:**
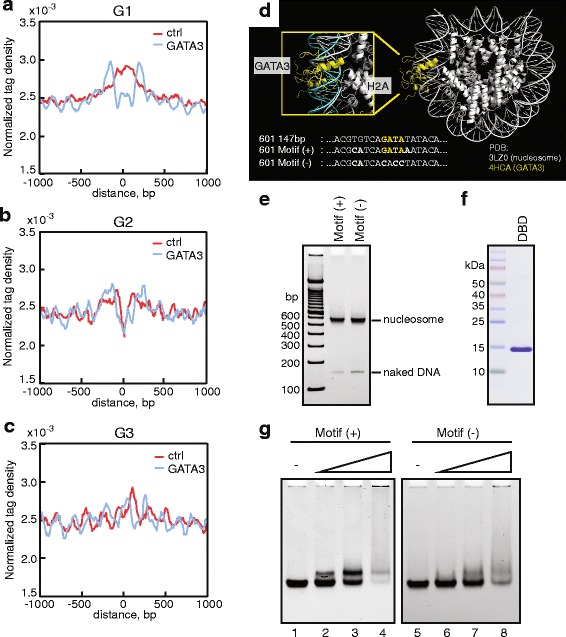
GATA3 induces nucleosome reorganization by targeting nucleosomal DNA. Metagene plots of normalized tag density of MNase-seq in G1 (**a**), G2 (**b**), and G3 (**c**) categories. Tag densities were normalized by the total counts in a +/- 1 kb window centered on the midpoint of GATA3 binding sites, then smoothed with a moving average (N  = 5). **d** The model of GATA3 bound nucleosome. GATA3 DBD and nucleosome core particle are represented in yellow and white, respectively. In the zoomed image, double-stranded DNA co-crystallized with GATA3 DBD is highlighted in cyan. The modified sequences are also indicated below. **e** Reconstituted nucleosome by salt dialysis method. The GATA consensus motif positive and negative DNAs were used to reconstitute mononucleosomes. **f** Purified recombinant GATA3 DBD. Purified proteins was analyzed by SDS-PAGE followed by staining with Coomassie. **g** Gel shift assay showing nucleosome binding of GATA3 DBD. The consensus motif containing nucleosome was used in lanes 1 to 4. The motif-mutated nucleosome was used in lanes 5 to 8. Nucleosomes (at a final concentration of 0.2 μM) were incubated with GATA3 DBD for 30 min at room temperature, and analyzed on native-polyacrylamide gel. The protein concentration of GATA3 DBD is as follows: 0 μM (lanes 1, 5), 0.5 μM (lanes 2, 6), 1 μM (lanes 3, 7), and 2 μM (lanes 4, 8)

To further explore this model, we determined the nucleosomal architecture of GATA3-binding sites by MNase-seq before and after GATA3 expression. As expected, pre-open chromatin regions (G2) exhibited the typical chromatin structure frequently observed at TF-binding sites (Fig. 3b). The center of the GATA3 peak has lower nucleosome density (so called nucleosome-depleted regions) flanked on each side by higher nucleosome density; this structure was conserved after the introduction of GATA3 (Fig. 3b). Notably, in the absence of GATA3, target sites in G1 presented high nucleosome density over the GATA3 peak, suggesting GATA3 may target regions occupied by nucleosomes (Fig. 3a). At these loci, GATA3 binding led to eviction of the nucleosome positioned over the GATA3 binding site with concomitant production of phased nucleosomes flanking the GATA3 site - similar to the profile observed in G2. Binding sites in G3 displayed a regular array of nucleosomes in the absence of GATA3. At these loci, GATA3 expression led to nucleosome sliding such that the nucleosome array, while still present, was shifted relative to DNA sequence (Fig. 3c).

The cellular data above suggested that GATA3 may bind to nucleosomes within regions of closed chromatin. To test this hypothesis, we first generated a model structure of a GATA3-bound nucleosome by utilizing crystal structure information [30, 47] for the nucleosome and for the GATA3 DNA binding domain (DBD) (Fig. 3d). In the resulting model, the GATA3-DNA complex was aligned by the ‘GATA’ sequence found within the 601-nucleosome positioning sequence [48]. An appreciable structural barrier to binding was not observed in this model. Therefore, we reconstituted nucleosomes on 601-based DNAs with or without the consensus GATA motif using recombinant human histones (Fig. 3d, e). We also purified the DNA binding domain of GATA3 from engineered bacteria, and performed gel shift analyses (Fig. 3f, g). In the presence of its binding motif, GATA3 displayed discrete shifted bands, suggesting specific binding to the nucleosomal target (Fig. 3g, lanes 1 to 3). However, in the absence of the motif, the binding affinity was significantly reduced and appeared to be non-specific (Fig. 3g, lanes 5 to 7). In both cases, the use of excess amounts of protein resulted in non-specific nucleosome binding (Fig. 3g, lanes 4 and 8). We thus concluded that GATA3 can interact productively with its target recognition sequence within the context of a nucleosome, consistent with function as a pioneer.

### Transactivation domain is required for GATA3-mediated chromatin remodeling

**Fig. 4:**
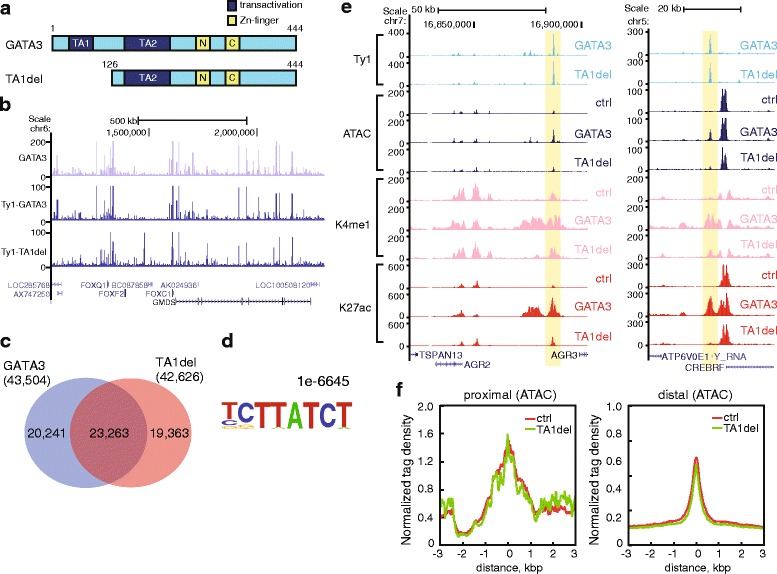
N-terminal truncation mutant binds but fails to open chromatin. **a** Schematic representation of wild-type GATA3 and N-terminal domain lacking (TA1del) mutant. **b** Genomic distribution of TA1del mutant. ChIP-seq with antibody against GATA3 or Ty1 is shown in purple or dark blue, respectively. ChIP-seq signals in wild-type GATA3 expressing cells were represented in the top and middle panels, while ChIP-seq signals in TA1del mutant expressing cells were shown in the bottom panel. **c** Venn diagram showing the peak overlap between wild-type GATA3 and TA1del mutant. **d** The top motif identified by *de novo* motif analysis is represented with *P* values. ChIP-seq peaks of TA1del mutant were analyzed by HOMER software. **e** Representative genomic loci showing that TA1del mutant expressing cells do not exhibit increased levels of ATAC-seq, H3K4me1 and K27ac signals as compared with wild-type GATA3 expressing cells. **f** Metagene profiles of normalized ATAC-seq signals showing the average tag density in TSS-proximal or -distal (>1 kb) peak flanking regions. The x-axis indicates the distance from the midpoint of TA1del mutant ChIP-seq peaks

The biochemical experiments with purified proteins suggested the DNA-binding domain was sufficient to bind to nucleosomal DNA. To identify the functional domain(s) required for GATA3-mediated chromatin remodeling, we established MDA-MB-231 cells expressing a mutant version of GATA3 lacking one of the previously-defined transactivation domains [49] (Fig. 4a). The average expression level of this mutant was equivalent to wild-type GATA3 (Additional file 1: Figure S4A, S4C, S4D). Importantly, cells expressing this mutant version of GATA3 fail to reprogram to an epithelial phenotype at the cellular and molecular level (Additional file 1: Figure S4B, S4D). We then defined the genome-wide distribution of this mutant by ChIP-seq, using the Ty1 epitope tag. We observed that ChIP-seq signals of wild-type and mutant versions of GATA3 were similar (Fig. 4b; Additional file 1: Figure S4E, S4F). Although a number of differential binding peaks were detected, many enriched binding sites were conserved between wild-type GATA3 and the TA1 mutant (Fig. 4b, c; Additional file 1: Figure S4G). We have limited subsequent analyses to those peaks found in both datasets. The consensus GATA3 motif was also enriched at loci bound by the TA1 mutant (Fig. 4d). Surprisingly, remodeling of chromatin structure and induction of histone modification was virtually abolished by deletion of the amino-terminal transactivation domain (Fig. 4e, f, Additional file 1: Figure S1F). We performed peak classification using parameters identical to those used in Fig. 2a, finding only 327 sites (<1 %, 11,035 sites in the case of wild-type) which were categorized as increasing accessibility following mutant GATA3 expression (Additional file 1: Figure S5).

**Fig. 5:**
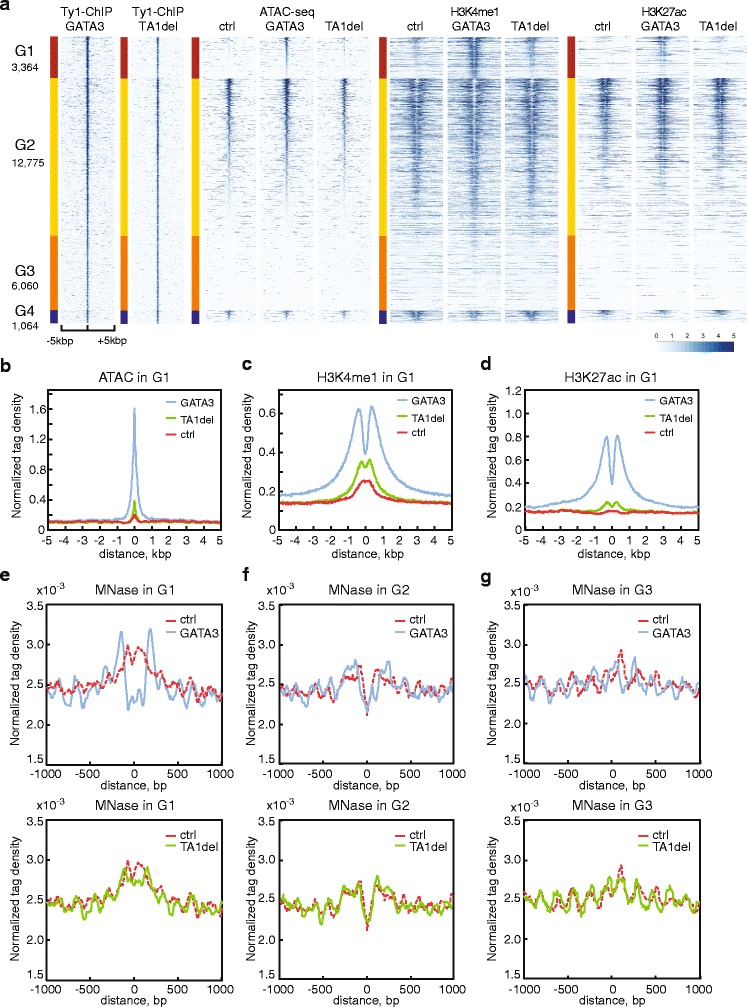
Transactivation domain is required for chromatin reprogramming. **a** Read density heatmaps showing the signal enrichment of Ty1 ChIP-seq, ATAC-seq, H3K4me1, and K27ac ChIP-seq in control, wild-type, and mutant GATA3 expressing cells. The same classification was utilized, but only the sites where both wild-type and mutant were localized are represented. The number of peaks in each category is reported. Each row indicates a 10 kb window centered on a GATA3 binding site. The scale of read density after normalization is indicated at the bottom right. Metagene profiles of normalized ATAC-seq (**b**), H3K4me1 ChIP-seq (**c**), and H3K27ac ChIP-seq (**d**) in G1 are shown for the comparison of the average signal levels in control, wild-type GATA3, and TA1del mutant expressing cells. Metagene profiles in the other groups are shown in Additional file 1: Figure S5. Metagene profiles of normalized MNase-seq signal density in G1 (**e**), G2 (**f**), and G3 (**g**) are shown, respectively. Top panels indicate the comparison between control and wild-type GATA3 expressing cells. Bottom panels indicate the comparison between control and TA1 del mutant expressing cells. Only overlapped peaks between wild-type and mutant GATA3 were used for the metagene analyses (**b**-**g**)

**Fig. 6:**
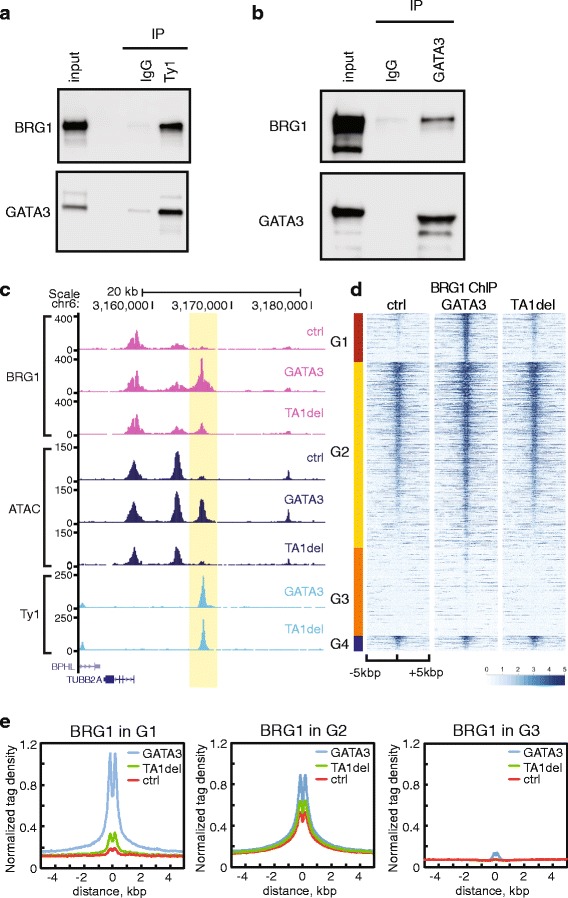
BRG1 interacts with GATA3, and is recruited at GATA3-bound chromatin domains. **a** Ty1-GATA3 was immunoprecipitated from MDA-MB-231 nuclear extracts with Ty1 antibody. Immunoblots were performed with the indicated antibodies. **b** Endogenous GATA3 was immunoprecipitated from T47D nuclear extracts with GATA3 antibody. Immunoblots were performed with the indicated antibodies. **c** Representative genomic locus showing the BRG1 enrichment in control, wild-type, and mutant GATA3 expressing cells. The highlighted region displays increased signals of BRG1 ChIP-seq in wild-type GATA3 expressing cells. **d** Read density heatmaps showing the signal enrichment of BRG1 ChIP-seq signals. The same classification and common peaks used in Fig. 6a are represented. **e** Metagene profiles of normalized BRG1 ChIP-seq tag density in G1, G2, and G3 are shown

In order to compare wild-type and mutant versions of GATA3, we focused on binding sites where both wild-type and the mutant were co-localized finding 23,263 peaks were shared between the two datasets (Fig. 4c). We observed that the TA1 mutant version of GATA3 failed to induce accessibility at loci where wild-type GATA3 did (Fig. 5a, b, Additional file 1: Figure S5B-D). In addition, mutant GATA3 failed to induce local histone modifications at these loci (Fig. 5c, d, Additional file 1: Figure S5E-J). We also noted that the ChIP-seq tag density of the mutant in category G1 (but not G2 and G3) was decreased when compared to wild-type GATA3 (Additional file 1: Figure S6), and was nearly identical to the density of the mutant in G3 (Additional file 1: Figure S6D). Further, the number of loci at which both wild-type and mutant versions co-localized was substantially lower in the pioneer category G1 as compared to categories G2 and G3 in which accessibility is not impacted by GATA3 binding (Figs. 2a and 6a). Although this truncation mutant possesses an intact DNA-binding domain, these data suggested that the mutant is impaired in pioneer activity. To further explore local chromatin architecture in wild-type versus mutant GATA3 expressing cells, we compared the MNase-seq signals in each cell line. The digestion patterns in the mutant-expressing cells were similar to those in control cells (Fig. 5e-g). The nucleosome occupancy at the GATA3 binding sites in category G1 was largely unchanged in the presence of the TA1 mutant, indicating a failure in nucleosome eviction (Fig. 5e). Further, at loci within category G3, the TA1 mutant failed to initiate nucleosome sliding around the center of the peak (Fig. 5g). These results suggested that the N-terminal transactivation domain (1-125aa) of GATA3 is critical for GATA3-mediated chromatin remodeling and the induction of histone modifications.

### GATA3 interacts with BRG1, a chromatin remodeling factor in solution and on chromatin

The inability of mutant GATA3 to remodel chromatin suggested that either the transactivation domain has chromatin remodeling activity similar to the carboxyl-terminal domain of FOXA1 [11], or that this region functions as a platform to recruit co-factors. Enrichment analysis of genes assigned to the G1 group identified the BRG1/SMARCA4 chromatin remodeling factor as a candidate co-factor (Additional file 1: Table S4). BRG1 is also known to function with the other GATA family proteins such as GATA1 and GATA4 [23, 50, 51] and is characterized by its ability to evict nucleosomes [52–54]. Therefore we asked whether BRG1 and GATA3 had biochemical interactions. We prepared conventional nuclear extracts from MDA-MB-231 cells expressing GATA3. Co-immunoprecipitation analysis indicated solution interactions between GATA3 and BRG1 in the MDA-MB-231 context (Fig. 6a). This interaction was further confirmed by co-immunoprecipitation of the endogenous proteins from T47D cells (Fig. 6b). Accordingly, we explored BRG1 accumulation at GATA3 binding sites in our MDA-MB-231 system using ChIP-seq for endogenous BRG1. We observed consistent enrichment of BRG1 at GATA3 binding sites in cells expressing wild-type, but not mutant, GATA3 at loci where GATA3 functions as a pioneer and evicts nucleosomes (Fig. 6c-e; Additional file 1: Figure S6E, S6F). These data demonstrate that BRG1 recruitment correlates with GATA3-dependent nucleosome eviction in a manner dependent on local context and on the GATA3 amino terminal activation domain.

## Discussion

Information content embedded in eukaryotic chromosomes modulates transcriptional output, influencing the transcriptional patterns integral to biological processes. A critical unit of this information is the TF-DNA complex, which can modulate the gene expression network at multiple levels. TFs receive instruction from local DNA sequence, reading the DNA backbone and the functional groups protruding into the major and minor grooves to recognize specific binding sites in genomic DNA. In eukaryotic nuclei, this paradigm is complicated by the packaging of DNA into chromatin which regulates access to the chemical moieties on DNA that drive productive TF-DNA recognition events. Integral to the elaboration of cell type and specialized function is a requirement for certain TFs to penetrate previously inaccessible chromatin leading to new patterns of transcriptional output.

In this report, we provide detailed analysis of GATA3-mediated chromatin reprogramming in the context of MET in a system relevant to breast cancer; we observed several key outcomes. At approximately 25 % of bound loci (category G1), GATA3 has the capacity to pioneer new binding sites in previously inaccessible chromatin that features high nucleosome occupancy over the GATA motif prior to GATA3 expression. At approximately 45 % of bound loci (category G2), GATA3 binds to a motif located within accessible chromatin where it appears to modestly increase the width of the accessible region. Surprisingly, we observed another 25 % of bound loci (category G3) where GATA3 occupancy is clearly detected by ChIP-seq, but where the local chromatin architecture remains refractory to transposition following GATA3 binding. Biochemical experiments suggested that protein-DNA interactions at the latter category of bound loci – where GATA3 binds to inaccessible chromatin and fails to remodel local architecture to a transposition-permissible state – were fundamentally different than the other two major categories. GATA3 could be functioning to alter chromatin and transcriptional status at both category G1 and G2, however category G2 sites were already open and did not exhibit drastic differences in chromatin architecture following expression of GATA3. Because of this limitation, we focused on the functional properties of binding events in category G1 and G3. We interpret the data to support a model for GATA3 interaction with chromatin. We envision that the protein scans the chromatin fiber for consensus binding motifs where the chemical moieties involved in productive interaction are positioned relative to the nucleosome surface in a manner providing access for interrogation by protein. Recognition of a productive binding site within a nucleosomal context engenders formation of a GATA3/nucleosome complex, a species we are able to recapitulate *in vitro* (Fig. 3d-g). At a subset of such sites, GATA3 is able to initiate nucleosome eviction over the center of the binding site with subsequent generation of flanking arrays of positioned nucleosomes bearing the hallmarks of an active enhancer (Fig. 2). Such sites are characterized by the GATA3-dependent generation of chromatin accessible to the structural probes sampled here, and by acquisition of active enhancer histone marks. At other sites, GATA3 is able to productively bind as measured by ChIP-Seq. In contrast to category G1 loci, these category G3 sites are characterized both before and after GATA3 binding by a nucleosome array which slightly slide relative to DNA sequence following expression of GATA3. These sites exhibit no evidence of nucleosome eviction over the binding site and do not generate accessible chromatin. We propose that association of co-factors to a GATA3/nucleosome complex differs in a context-dependent manner and that the nature of specific co-factors fundamentally alters outcome. At pioneering loci, the action of co-factors including the SWI/SNF complex alters the protein/substrate interaction, leading to nucleosome eviction and generation of accessible chromatin (Fig. 2e, f). At category G3, other co-factors lead to nucleosome sliding, repositioning an array of nucleosomes relative to DNA sequence without generation of accessibility. In both instances, alterations in local chromatin are dependent on the presence of the activation domain.

The model proposed here envisions two distinct steps to pioneering activity by GATA3 – binding followed by local remodeling – and differs from that explaining the pioneering activity of FOXA1. Elegant biochemical and cellular data suggest that FOXA1 has the intrinsic capacity to elicit local perturbation of chromatin structure without the involvement of additional co-factors [11]. Pioneering activity by GATA3, on the other hand, correlates with the capacity to recruit the mammalian SWI/SNF chromatin remodeling complex and requires the presence of the transcriptional activation domain (Figs. 5 and 6). While local remodeling by FOXA1 appears inextricably linked to productive binding, GATA3 appears to require co-factors for this function. A critical question arises as to why GATA3 binding leads to distinct outcomes at different loci despite the observation of a biochemical interaction between GATA3 and BRG1. It is possible that this interaction alone is not sufficient to drive the initiation of nucleosome eviction at all loci; it is also conceivable that local architectural constraints influence interaction with other co-factors (Additional file 3: Table S2). Whatever the proximate cause, the differences in local nucleosome architecture around GATA3 binding sites prior to GATA3 expression at loci that differ in outcome (nucleosome sliding versus eviction) (Fig. 3b, c) suggest architectural constraints may distinguish the two types of events. Architectural influences of DNA, and by extension chromatin, on outcomes of TF mediated events have been elegantly documented in the case of the glucocorticoid receptor [55].

GATA3 has been identified as one of the most frequently mutated genes in breast cancer [35, 56]. GATA3 mutations accumulate in a striking pattern at the C-terminal region of the protein including the second zinc finger [56, 57]. Mutations in the DNA-binding domain have been shown to affect chromatin binding activity and motif recognition [43] and are likely to lead to alterations in genomic distribution. The outcomes of mutations at the far carboxyl terminus of GATA3, an abundant class of cancer-specific mutants, are currently understudied. Because most cancer-derived mutants have an intact N-terminal domain, their abnormal distribution may induce inappropriate pioneering activity leading to reprogrammed gene expression. It is also conceivable that the cancer-specific mutations in GATA3 alter co-factor recruitment and interaction (either positively or negatively) leading to novel or impaired pioneering functions. In luminal breast cancer cells, GATA3 is known to functionally interact with the TFs FOXA1 and estrogen receptor-α [28, 58], leading to the potential for GATA3 mutations to alter the transcriptional network of these factors. Given the capacity of GATA3 to catalyze cellular reprogramming relevant to cancer biology, characterization of the impact of cancer-specific mutations in this pioneering TF represents a field or enquiry with major clinical ramifications.

## Conclusions

Our data provide new experimental evidence that GATA3 functions as a pioneer factor during cellular reprogramming using the MET of breast cancer cells as a model. Dissection of chromatin features during this process reveals key mechanistic features required to generate active chromatin structure: binding to a nucleosomal locus in the appropriate context and transactivation domain dependent co-factor recruitment. These findings provide novel insights into the mechanism of *de novo* enhancer establishment by a pioneer TF.

## Methods

### Generation of stable cell line

Lentiviruses were produced by transient transfection of 293T cells with pHAGE, psPAX2, and pMD2.G plasmids. psPAX2 and pMD2.G were gifts from Didier Trono (Addgene plasmid #12260, #12259). The virus-containing medium and polybrene (at a final concentration of 8 μg/mL) were added to MDA-MB-231 cells. Infected cells were selected with 0.5 μg/mL puromycin. Only early passage cells (passages 3 to 7) were used in all experiments. Cells expressing either GFP or the tag alone (including the TEV protease recognition site) were used as a control cell line.

### Nucleosome binding assay

The reconstituted nucleosomes and GATA3 DBD were incubated at room temperature for 30 min in the reaction buffer containing 30 mM Tris-HCl (pH 7.5), 120 mM NaCl, 25 mM KCl, 4 % glycerol, 1.5 % Ficoll 400, 0.4 mM zinc sulphate, and 0.1 mM EDTA. The complex and free nucleosomes were separated on 6 % native-polyacrylamide gel, and visualized by ethidium bromide staining.

### ATAC-seq sample preparation and data analysis

The sequence library for ATAC-seq was prepared as previously described [44] with several modifications (see Additional file 1: supplementary methods). Reads were mapped to hg19 genome using Bowtie 0.12.8 [59] with the same parameter used in [44]. Only non-duplicate reads were used for the subsequent analysis. The offset parameters suggested by Buenrostro *et al.* were applied to each mapped read [44]. Reads from three biological replicates were then merged for use in all subsequent analysis.

### MNase-seq

Native mononucleosomes were prepared as previously described [60] with several modifications (see Additional file 1: supplementary methods). Briefly, cells were treated with a hypotonic buffer for 15 min on ice. The isolated chromatin was digested with 4 U of MNase at 37 °C for 4 min. Digested chromatin was homogenized by passing through a 27G ½ needle (8 strokes), and the debris was removed by centrifugation. DNA was purified using a spin column. Mononucleosomes were separated by agarose gel electrophoresis, and purified by a QIAGEN gel extraction kit. Sequencing libraries were prepared by NEXTflex Rapid DNA-Seq kit. Reads were mapped to hg19 genome, and duplicate reads were removed using MarkDuplicates.jar from picard-tools-1.107 package (http://broadinstitute.github.io/picard/). All paired-end reads were converted to a single fragment for the metagene plot analysis.

### Chromatin immunoprecipitation (ChIP) for histones and BRG1

ChIP experiments were performed as previously described [61] with the following modifications. Cells were fixed with 1 % formaldehyde either at room temperature for 10 min (histone ChIP) or at 37 °C for 20 min (BRG1 ChIP), and quenched with glycine. For BRG1 ChIP, fixed cells were further treated with a hypotonic buffer. Cell pellets were resuspended with lysis buffer A (20 mM Tris-HCl pH 8.0, 2 mM EDTA, 0.5 mM EGTA, 0.5 mM PMSF, 5 mM sodium butyrate, 1 % SDS and protease inhibitor cocktail) for histone ChIP or lysis buffer B (same as buffer A, except 0.1 % SDS) for BRG1 ChIP. Chromatin was fragmented by sonication with Bioruptor (histone ChIP), or Covaris S220 (BRG1 ChIP), then diluted to adjust the SDS concentration to 0.1 % or 0.05 %. Immunoprecipitation was performed with indicated antibodies (Additional file 1: Table S5). Dynabeads Protein A and G were used to capture antibodies, and beads were washed with 1 mL of low salt (20 mM Tris-HCl pH 8.0, 150 mM NaCl, 2 mM EDTA, 1 % Triton X-100, 0.1 % SDS), high salt (same as low salt buffer, except 500 mM NaCl), and LiCl buffer (Tris-HCl pH 8.0, 250 mM LiCl, 2 mM EDTA, 1 % NP-40, 1 % (wt/vol) sodium deoxycholate). Eluted DNA was treated with RNase at 65 °C, followed by the incubation with proteinase K. DNA was purified by AMPure XP. Histone and BRG1 ChIP experiments were conducted with two and three biological replicates, respectively (Additional file 1: Table S5).

### ChIP for GATA3

Cells were treated with CSK buffer 2 (same as described above, except containing 1 mM EGTA, 2 mM EDTA, 1 mM PMSF, and protease inhibitor cocktail) for 5 min on ice or at 37 °C. After centrifugation at 100 g for 5 min at 4 °C, nuclear pellets were washed with CSK buffer 3 (same as CSK buffer1, except that Triton X-100 was omitted). After centrifugation, nuclei were resuspended with CSK buffer 3, then an equal volume of 2 % formaldehyde containing CSK buffer 3 was added to the solution, followed by the incubation at 4 °C for 15 min. The fixation was quenched by addition of glycine, and the fixed nuclei were washed twice with CSK buffer 3. The nuclei were lysed with 0.5 % SDS containing lysis buffer A, and chromatin was fragmented by sonication with Bioruptor. The fragmented chromatin was diluted with nine-fold volume of IP buffer (20 mM Tris-HCl pH 8.0, 150 mM NaCl, 2 mM EDTA, 10 % glycerol, 0.5 % Triton X-100, 0.5 mM PMSF, 5 mM sodium butyrate, and protease inhibitor cocktail). Immunoprecipitation was performed as described above with two biological replicates (Additional file 1: Table S5). Homemade GATA3 antibody (prepared in Wade laboratory) was used for ChIP experiments [43], and Ty1 antibody was prepared in Stunnenberg laboratory [62].

### ChIP-seq analysis

Reads were filtered based on a mean base quality score >20, and mapped to hg19 genome using Bowtie 0.12.8 [59]. In order to minimize PCR amplification bias, duplicate reads were removed using MarkDuplicates.jar from picard-tools-1.107 package. Reads from the biological replicates were merged for use in all subsequent analysis. All paired-end reads were converted to a single fragment for the metagene plot analysis and visualization on the UCSC Genome Browser. After normalization to the indicated counts (Additional file 1: Table S6), bigWig files were generated to visualize the genomic coverage.

### Peak call and motif analysis

Peak calling and motif analyses were performed using HOMER v4.1 with default parameters [63]. Motif length 8, 10, and 12 bases were used for *de novo* motif analysis. Based on the distance from the midpoint of a peak to the nearest RefSeq transcription start site, we defined all peaks as either TSS-proximal (distance: less than or equal to 1 kb) or distal (distance: more than 1 kb) peaks.

### GATA3 peak classification based on ATAC-seq

EdgeR [64] was used to divide the GATA3 peaks into three groups (signal stronger: G1, signal conserved, and signal reduced: G3) based on the signal changes of ATAC-seq before and after GATA3-induced epithelial transition. After normalization to 30 million total reads, ATAC-seq read counts in the 200 bp window centered on GATA3 peaks were collected. The following parameters were used for the peak classification: FDR <0.1 and fold change >2. The signal conserved group was further subclassified into signal positive (G2) and negative (G3) groups based on a threshold of 20 counts in the +/- 1 kb region flanking the peak midpoint. In the heatmaps (Figs. 2a, 5a, and 6d, Additional file 1: Figure S5A), the peaks are ordered by decreasing weighted ATAC signal within each of the four peak classes.

### Statistical analysis

Box plots were generated by with bwplot (lattice R package; for randomly selected GATA motif containing genomic regions) or Prism 6 (GraphPad Software). Mann-Whitney test was used to compare data groups. Asterisk indicates *P* value <0.0001.

### Availability of supporting data

The data discussed in this publication have been deposited in NCBI’s Gene Expression Omnibus [65] and are accessible through GEO Series accession number GSE72141 (http://www.ncbi.nlm.nih.gov/geo/query/acc.cgi?acc=GSE72141).

### Ethics approval

Ethical approval was not needed for this study.

## Additional files

Additional file 1:
**is a document containing the supplementary figures, legends, tables, and methods.** (PDF 13200 kb) Additional file 2:
**is a table listing associated genes in each ATAC-seq category.** (XLSX 645 kb) Additional file 3:
**is a table listing differentially expressed TFs.** (XLSX 64 kb)
