# Supplementary material for: GATA3-dependent cellular reprogramming requires activation-domain dependent recruitment of a chromatin remodeler
Source: Genome Biol. 2016 Feb 27;17:36. doi: 10.1186/s13059-016-0897-0 (PMC4769547; doi:10.1186/s13059-016-0897-0)
Supplement: Additional file 1: — is a document containing the supplementary figures, legends, tables, and methods. (PDF 13200 kb) [file 13059_2016_897_MOESM1_ESM.pdf]

Legends to Supplemental Figures

Supplemental Figure 1. GATA3 expressing cells display chromatin reprogramming along with epithelial phenotype at cellular and molecular levels

(A) Immunoblots were performed with the indicated antibodies. Whole cell extracts ($1x10^5$ cells/lane) were resolved by SDS-PAGE. T47D is a GATA3 positive luminal breast cancer cell line. β-actin was used as an internal loading control. (B) Representative images of control (top) and GATA3 expressing (bottom) MDA-MB-231 cells. The scale bar indicates 100 μm. (C) Heatmap showing the differentially expressed genes (DEG). Each condition has three biological replicates. The thresholds used to define DEG are indicated. 1449 up-regulated genes and 790 down-regulated genes were identified. Epithelial (top) and mesenchymal (bottom) marker genes are shown in the box. (D) Reproducibility of GATA3 ChIP-seq. The scatter plot shows normalized signal intensity at GATA3 peaks (+/- 100 nt from peak midpoint) between two biological replicates. Each data set was normalized to 10 million mapped fragments, and the image was generated with smoothScatter (R package). The red line indicates the trend line calculated by linear regression, and the black line indicates the diagonal line (y=x). Pearson's r value is indicated at the top. (E) Venn diagram showing the overlap of GATA3 ChIP-seq peaks in MDA-MB-231, MCF7, and T47D cells. (F) UCSC genome browser shots showing representative genomic loci where ATAC-seq signals were increased in MDA231-GATA3 cells compared to control cells. (G) The box-and-whisker plots showing the normalized counts of ATAC-seq signals at randomly selected genomic loci. Each data set was normalized to 30 million mapped fragments. 1,000 genomic regions, which contain the GATA3 consensus motif (WGATAR), were randomly selected, and the mapped read counts (+/- 100bp centered on the motif) were collected. In order to avoid the potentially unmappable regions, we excluded any regions where the read count from all 3 datasets was zero. The top and bottom whiskers indicates 2th and 98th percentile, respectively. The black and red dots indicate the median and mean. The p-values are indicated at the bottom.

Supplemental Figure 2. Chromatin characterization of GATA3 binding regions

Metagene profiles of normalized ATAC-seq (A-C), H3K4me1 ChIP-seq (D-F), and H3K27ac ChIP-seq (G-I) are shown.

(J) Metagene profiles of normalized GATA3 ChIP-seq tag density in each group are represented.

(K) Metagene plots of normalized tag density of GATA3 ChIP-seq in G2 performed with or without thermal treatment at 37 ^{o}C.

(L) Box-and-whisker plots showing the fold changes of GATA3 ChIP-seq signals between "on ice" and 37 ^{o}C thermal treatment. Each data set was normalized to 27 million mapped fragments, then the fold change in each peak was calculated. The top and bottom whiskers show 5th and 95th percentile, respectively. The horizontal line and cross mark the median and mean. *$p < 0.0001$, Mann-Whitney test.

(M) Chromatin conformation of GATA3 binding sites in MCF7. Read density heatmaps showing the normalized signal intensity of GATA3 ChIP-seq, and DNase-seq in MCF7 cells. (N) Read density heatmaps showing the signal intensity of GATA3 ChIP-seq, and DNase-seq in T47D cells. GATA3 binding peaks were subdivided into the DNase-seq peak positive (red) or negative groups (yellow) based on overlap of the GATA3 peak region (+/- 200 nt from peak midpoint) with DNase-seq peaks as defined by ENCODE. The number of peaks in each category is reported below the group label.

Supplemental Figure 3. Putative GATA3 target genes in G1 associated with functional outcome: epithelial transition

(A-D) Relative gene expression associated with each GATA3-peak group. Peak-to-gene assignment was carried out based on the distance from peak to nearest TSS, with maximum distance 5 kb (A, C) or 50 kb (B, D). . Fold changes were calculated by DESeq2. More than 1.5 fold change was considered up-regulated, and less than -1.5 fold change was considered down-regulated. Genes that have DESeq2 basemean value $\leq$ 1 were excluded. Total number of genes in each group is indicated in parentheses. In the panel C and D, the fold changes in each group are shown as a box-and-whisker plot. The top and bottom whiskers show 10th and 90th percentile, respectively. The horizontal line marks the median. *$p < 0.0001$, Mann-Whitney test.

(E) Time course analysis of G1-related gene expression measured by qPCR. RNAs were collected at each time points post-infection. The experimental data are presented as means +/- standard deviations from three biological replicates. The ΔΔCt method with TBP as reference gene is used for calculation of relative gene expression (*$p < 0.03$, unpaired two-tailed t test).

(F, G) Canonical pathway analysis by IPA software (QIAGEN). The closest genes within 50 kb were used for the pathway analysis. Top 10 significant canonical pathways predicted from G1 (F) and G3 (G) associated genes are listed.

Supplemental Figure 4. Transactivation domain lacking mutant fails to induce mesenchymal-to-epithelial transition

(A) Immunoblots were performed with Ty1 antibody. Whole cell extracts were resolved by SDS-PAGE. Vinculin was used as an internal loading control. (B) Representative images of TA1del mutant expressing MDA-MB-231 cells. The scale bar indicates 100 μm. (C) Immunostaining was performed with Ty1 antibody. The nuclear DNA was stained with DAPI. (D) Candidate gene expression measured by qPCR. The experimental data are presented as means +/- standard deviations from three biological replicates. The ΔΔCt method with TBP as reference gene is used for calculation of relative gene expression.

(E, F) Reproducibility of Ty1 ChIP-seq. The scatter plots represent normalized signal intensity at GATA3 peaks (E) and TA1del mutant peaks (F) (+/- 100 nt from peak midpoint) between two biological replicates. Each data set was normalized to 10 million mapped fragments, and the smoothed plots were generated with smoothScatter. The red line indicates the trend line calculated by linear regression, and the black line indicates the diagonal line (y=x). Pearson's r values are indicated at the bottom.

(G) Comparison of chromatin binding properties between GATA3 and TA1del mutant. Read density heatmaps showing the normalized signal intensity (+/- 100 nt from peak midpoint) from each Ty1 ChIP-seq data. Wild-type and mutant GATA3 binding peaks were subdivided into shared peaks (green), wild-type unique peaks (red), and mutant unique peaks.

Supplemental Figure 5. Functional properties of TA1del mutant on chromatin

(A) Read density heatmaps showing the signal intensity of Ty1 ChIP-seq, ATAC-seq, H3K4me1 and H3K27ac ChIP-seq in either control or TA1del mutant expressing cells. Classification from G1 to G4 was carried out based on ATAC-seq signal changes between control and TA1del mutant expressing cells. The number of peaks in each category is represented. Each row indicates a 10 kb window centered on the mutant binding site. The scale of read density after normalization is indicated at the bottom right.

(B-J) Metagene profiles of normalized ATAC-seq (B-D), H3K4me1 ChIP-seq (F-G), and H3K27ac ChIP-seq (H-J) are shown for the comparison of the average signal levels in each group. The common binding sites between wild-type and mutant were used for the plots.

Supplemental Figure 6. Characteristic differences between wild-type and TA1del mutant GATA3

(A-C) Chromatin binding affinity of TA1del mutant. Metagene profiles of normalized Ty1 ChIP-seq tag density in G1 (A), G2 (B), and G3 (C) binding categories are represented. Blue line profiles indicate ChIP-seq data from GATA3 expressing cells, and green line profiles indicate ChIP-seq data from TA1del mutant expressing cells.

(D) Box-and-whisker plot showing the distribution of normalized Ty1 ChIP-seq tag density in wild-type or TA1del mutant GATA3 expressing cells. The counts (+/- 100bp centered on the common binding sites) were collected, and normalized to 20 million mapped fragments. The top and bottom whiskers show 5th and 95th percentile, respectively. The horizontal line marks the median. *$p < 0.0001$, Wilcoxon signed rank test.

(E, F) Wild-type GATA3 and TA1del mutant show differential correlation with BRG1 distribution on chromatin. The normalized signal intensity of Ty1 ChIP-seq was plotted against that of BRG1 ChIP-seq. Each data set was normalized to 10 million mapped

fragments, and the read counts in 1 kb regions centered on GATA3 peaks (E) or TA1del mutant peaks (F) were collected. The smoothed plots were generated with smoothScatter.

Supplemental Table 1. Associated gene list in each ATAC-seq category
Peak-to-gene assignment was carried out based on the distance from peak to nearest TSS, with maximum distance 50 kb. Fold changes were calculated by DESeq2. Genes that have DESeq2 basemean value $\leqq$ 1 were excluded.

Supplemental Table 2. Differentially expressed transcription factors.
The Uniprot database [1] was utilized to extract the differentially expressed TFs from the DEG list described in Figure S1C. Homer de novo motif analysis was performed to investigate the motifs of potential cofactors ($P < e\text{-}200$).

Supplemental methods

Antibody

Antibodies used for ChIP experiments are indicated in Table S5. GATA3 antibody from Cell Signaling Technology (D13C9), β-actin from abcam (ab8226) were used for immunoblotting. BRG1 (obtained from Dr. Archer) and Ty1 (obtained from Dr. Stunnenberg [2]) were used for immunoprecipitation.

DNA construction

The lentiviral vector (pHAGE) for wild-type and mutant GATA3 expression was kindly provided by Dr. Guang Hu (NIEHS/NIH). Human GATA3 and N-terminal deletion mutant (126aa-444aa) were amplified and cloned into pENTR/D-topo vector (Thermo Fisher Scientific). 3xTy1 tag and TEV protease recognition sequences were added at the N-terminus of each gene. The cloned genes were transferred into the destination vector by LR reaction (Thermo Fisher Scientific).

Cell culture

MDA-MB-231 and 293T cells were obtained from the ATCC, and grown in DMEM high-glucose medium with 10% FBS (Thermo Fisher Scientific) in 10% CO_2 at 37^oC.

Protein purification of GATA3 DNA-binding domain (DBD)

The DNA-binding domain (DBD) of GATA3 (261aa-371aa) was purified as previously described [3] with the following modifications. After Ni-NTA agarose column chromatography (QIAGEN), the His-tag was removed by digestion with thrombin protease. The GATA3 DBD was further purified by MonoS column followed by gel filtration using Superdex 75 column (GE Healthcare Life Sciences).

Nucleosome reconstitution

Recombinant histone octamers and the GATA3-DNA binding domain (GATA3 DBD) were prepared as described previously [3, 4] with modifications (See Supplemental experimental procedures). The purified histone octamers were mixed with 601 DNAs

(1:2 molar ratio of DNA:histone octamer), and the nucleosomes were reconstituted by the salt dialysis method as described in [4]. The modified 601 DNA sequences are listed in Table S7.

Model structure

Crystal structures of GATA3-DNA complex (4HCA) [5] and nucleosome core particle (3LZ0) [6] were used to generate a model structure of GATA3-bound nucleosome. The GATA3-DNA complex was aligned by the "GATA" sequence position found within the 601-nucleosome. The programs Coot [7] and PyMOL (The PyMOL Molecular Graphics System, Version 1.7.4 Schrödinger, LLC) were used for all the structural analyses.

Immunoblotting

Cells were lysed with sample loading buffer. $1x10^5$ cells were used for each lane. Extracted proteins were resolved on SDS-PAGE and immunoblotted using indicated antibodies. The chemiluminescent signals were detected and analyzed by the Odyssey Fc Imaging System (LI-COR).

Immunostaining

Cells growing on coverslips were fixed with 4% formaldehyde in 0.1% Triton X-100 containing PBS for 10 min at room temperature. Cells were washed with PBS, and blocked with 3% BSA in PBS-T (0.1% Tween 20) for 20 min. After wash with PBS, cells were incubated with anti-Ty1 antibody followed with three washed with PBS, and then incubated with the Alexa 488 fluor conjugated antibody (Thermo Fisher Scientific). The coverslips were mounted on slides with DAPI containing mounting medium (Vector Laboratories) and analyzed using a Zeiss Axiovert 200 M microscope.

Immunoprecipitation (IP) with nuclear extracts

Cells were lysed with hypotonic buffer A (10 mM HEPES-NaOH pH 7.9, 10 mM KCl, 1.5 mM $MgCl_2$, 340 mM sucrose, 10% glycerol, 0.5% Triton X-100, and protease inhibitor cocktail purchased from Thermo Fisher Scientific). The nuclei were

resuspended in a high salt buffer (20 mM HEPES-NaOH pH 7.5, 600 mM NaCl, 1.5 mM $MgCl_2$, 25% glycerol, 0.2 mM EDTA, 0.5 mM PMSF, 0.5 mM DTT, 0.1% Triton X-100, and protease inhibitor cocktail), and the nuclear proteins were obtained by ultracentrifugation. The salt concentration was adjusted to 150 mM by diluting with a dilution buffer (20 mM HEPES-NaOH pH 7.5, 1.5 mM $MgCl_2$, 10% glycerol, 0.2 mM EDTA, 0.5 mM PMSF, 0.5 mM DTT, 0.1% Triton X-100, and protease inhibitor cocktail). 450 μg or 1,500 μg of total protein was used as input for MDA-MB-231 or T47D IP experiments. Nuclear proteins were incubated with indicated antibodies, and captured by Protein G or Protein A agarose (EMD Millipore). Co-precipitated proteins were resolved by SDS-PAGE, and analysed by immunoblotting.

qPCR

Total RNA was isolated from cells using RNeasy Kit (QIAGEN). The cDNAs were synthesized by using iScript cDNA Synthesis Kit (Bio-Rad Laboratories). qPCR was performed using iQ SYBR Green Supermix and MyiQ instrument (Bio-Rad Laboratories). Ct values were normalized to TBP. Relative expression was calculated by delta-delta Ct method.

RNA-seq analysis

Library preparation and sequencing were performed by Expression Analysis on an Illumina platform. Reads were filtered based on a mean base quality score >20, and mapped to the hg19 reference genome with TopHat v2.0.4 (parameters --b2-sensitive --library-type fr-firststrand -g 10 -r 50 --mate-std-dev 50; other parameters default [8]). Mapped hits per gene were tabulated with HTSeq-count [9]. DESeq2 [10] was used for differential gene analysis, with thresholds of $|\log2FC| > 0.58$ and adjusted P-value < 0.05 for differentially expressed genes. The heatmap of differentially expressed genes is based on FPKM values as calculated by the CuffQuant and CuffNorm workflow (Cufflinks v2.2.1; [11]).

ATAC-seq sample preparation and data analysis

The sequence library for ATAC-seq was prepared as previously described [12] with the following modifications. 25,000 cells were incubated in CSK buffer 1 (10 mM PIPES pH 6.8, 100 mM NaCl, 300 mM sucrose, 3 mM $MgCl_2$, 0.1% Triton X-100) on ice for 5 min. 5 μl of Tn5 Transposase (total reaction volume: 25 μl) was used for the tagmentation. 8 total PCR cycles were performed, and amplified DNA fragments was purified with AMPure XP (1:3 ratio of sample to beads, Beckman Coulter).

The libraries were sequenced on HiSeq 2500 (Illumina) at the NIH Intramural Sequencing Center, MiSeq and NextSeq 500 (Illumina) at the NIEHS Epigenomics Core Facility. Reads were filtered based on a mean base quality score >20. After adapter trimming by Trim Galore! (Babraham Institute), reads were mapped to hg19 genome using Bowtie 0.12.8 [13] with the same parameter used in [12]. Only non-duplicate reads were used for the subsequent analysis. The offset parameters suggested by *Buenrostro et al* were applied to each mapped read. We confirmed the quality of each library and reproducibility of replicates by evaluating peak overlap ratios, signal-to-noise ratios, and fragment size distribution. Reads from three biological replicates were merged for use in all subsequent analysis.

MNase-seq

Native mononucleosomes were prepared as previously described [14] with the following modifications. $1x10^7$ cells were treated with a hypotonic buffer (10 mM Tris-HCl pH 7.5, 10 mM NaCl, 3 mM $MgCl_2$, 0.5% Triton X-100) for 15 min on ice. After centrifugation, the pellets were washed once with MNase digestion buffer (10 mM Tris-HCl pH 7.5, 15 mM NaCl, 60 mM KCl, 3 mM $CaCl_2$). The nuclei were resuspended with 500 μl of the MNase digestion buffer, and the chromatin concentration was determined by measuring the absorbance at 260 nm. 50 μg chromatin (100 μl) was digested with 4 U of MNase (Worthington Biochemical Corporation) at 37 ^oC for 4 min. The reaction was terminated by the addition of 100 μl of a stop buffer (10 mM Tris-HCl pH 7.5, 15 mM NaCl, 60 mM KCl, 25 mM EDTA). Digested chromatin was homogenized by passing through a 27G ½ needle (8 strokes), and the debris was removed by centrifugation at 20,000g for 5 min. DNA was purified using a Zymo Research spin column. Mononucleosomes were separated by agarose gel electrophoresis, and purified

by a QIAGEN gel extraction kit. Sequencing libraries were prepared by NEXTflex Rapid DNA-Seq kit using 100 ng input DNA. After 4 PCR cycles, adapter ligated mononucleosomal DNA (~250 bp) was excised, and purified by MinElute Gel Extraction Kit (QIAGEN). The libraries were sequenced on a NextSeq 500 (Illumina). Reads were filtered based on a mean base quality score >20, and mapped to hg19 genome using Bowtie 0.12.8. Duplicate reads were removed using MarkDuplicates.jar from picard-tools-1.107 package (http://broadinstitute.github.io/picard/). Reads from three biological replicates were merged for use in all subsequent analysis. All paired-end reads were converted to a single fragment for the metagene plot analysis.

ChIP-seq library preparation

The amount of input DNA, the sample preparation kit, and PCR cycles used in each library preparation are listed in Table S5. The libraries were sequenced on a Miseq or NextSeq 500 (Illumina).

Heatmap generation

Reads were collected in the 10 kb window centered on GATA3 peaks (bin size 20bp). After normalization to indicated reads (Table S6), heatmaps were generated with heatmap.2 (gplots R package). For the heatmap of differentially expressed genes, samples (columns) are clustered by hcluster (method=”euclidean”, link=”average”), and the genes (rows) are ordered alphabetically within differential expression category.

Metagene plot

Metagene plots were constructed by counting mapped fragments in 20bp bins flanking peak midpoints, averaging (i.e. dividing by the number of total peaks), and normalizing to 10 million mapped fragments. For MNase-seq metagene plots, 10bp bins were used and the data was normalized by total counts within +/- 2 kb; these plots were smoothed with a moving average trendline (N=5).

Peak to gene assignment

To assign GATA3 binding peaks to putative target genes, the distance from the center of each peak to closest TSS was calculated, and associated gene(s) were extracted. Gene models are from RefSeq, downloaded from the UCSC Genome Browser as of May 20, 2014. The maximum distance for gene assignments are indicated in each section.

DNase-seq analysis

The ENCODE DNase-seq data were utilized to generate heatmaps of MCF7 (GEO accession number: GSM1008565) and T47D cells (GEO accession number: GSM816673). Replicates were merged, then reads were collected in the 1 kb window centered on GATA3 peaks (bin size 20bp) (Adomas et al. 2014). The total mapped reads for each GATA3 ChIP-seq were normalized to 40 million total reads, and the DNase-seq reads were normalized to 100 million total reads. Heatmaps were generated with heatmap.2 (gplots R package).

Group	Total peaks	WGATAR motif containing peaks		Total WGATAR motifs
G1	11,035	8,705	78.9%	14,707
G2	19,585	13,210	67.5%	20,032
G3	11,458	9,494	82.9%	16,078
G4	1,426	862	60.4%	1,261

Supplemental Table 3. Consensus motif analysis

The number and percentage of peaks that contain at least one consensus motif are represented in column 3 and column 4, respectively. Total motifs found in each group are represented in column 5.

Upstream Regulator	Molecule Type	Predicted Activation State	Activation z-score	p-value of overlap
TP53	transcription regulator	Activated	3.753	5.38E-29
beta-estradiol	chemical	Activated	2.009	4.62E-24
tretinoin	chemical	Activated	2.247	5.84E-14
**SMARCA4**	transcription regulator	Activated	2.808	2.77E-11
doxorubicin	chemical drug	Activated	2.649	8.59E-11

Supplemental Table 4. Predicted upstream regulators

Top 5 upstream activators predicted by QIAGEN'S Ingenuity Pathway Analysis (IPA) are listed. SMARCA4 (BRG1) was identified as one of activators.

Data Type	antibody	Input DNA	Kit	PCR cycles	N	GEO accession
GATA3 ChIP-seq	Adomas et al., 2014 [3]	10 ng	TruSeq RNA Library Prep Kit v2 - Illumina	10	2	
Ty1 ChIP-seq	Menafra et al., 2014 [2]	10 ng	TruSeq RNA Library Prep Kit v2 - Illumina	10	2	
H3K4me1 ChIP-seq	abcam ab8895	50 ng	TruSeq RNA Library Prep Kit v2 - Illumina	9	2	
H3K27ac ChIP-seq	abcam ab4729	50 ng	TruSeq RNA Library Prep Kit v2 - Illumina	9	2	
BRG1 ChIP-seq	Prepared in Archer laboratory	2 ng	NEXTflex Rapid DNA-Seq Kit - BIOO SCIENTIFIC	12	3	
MNase-seq	N/A	100 ng	NEXTflex Rapid DNA-Seq Kit - BIOO SCIENTIFIC	4	3	
ATAC-seq	N/A	(25,000 cells)	Nextera DNA Library Preparation Kit - Illumina	8	3	

Supplemental Table 5. A list of sequencing libraries

The information of all libraries used in this study is listed. N indicates the number of biological replicates.

Data type	Normalized counts	
	Heatmap	Genome browser
GATA3 ChIP-seq	30,000,000	27,000,000
Ty1 ChIP-seq	30,000,000	20,000,000
H3K4me1 ChIP-seq	60,000,000	70,000,000
H3K27ac ChIP-seq	30,000,000	64,000,000
BRG1 ChIP-seq	60,000,000	71,000,000
ATAC-seq	30,000,000	29,000,000

Supplemental Table 6. Normalization for heatmap and visualization in genome browser

The fragment number used for each data analysis is listed.

	Sequence
601 motif positive (147 bases)	ATCGAGAATCCCGGTGCCGAGGCCGCTCAATTGGTCGTAGACAGCTCTAG<br>CACCGCTTAAACGCACGTACGGATTCTCCCCCGCGTTTTAACCGCCAAGG<br>GGATTACTCCCTAGTCTCCAGGCACGCATCAGATAAATACATCCGAT
601 motif negative (147 bases)	ATCGAGAATCCCGGTGCCGAGGCCGCTCAATTGGTCGTAGACAGCTCTAG<br>CACCGCTTAAACGCACGTACGGATTCTCCCCCGCGTTTTAACCGCCAAGG<br>GGATTACTCCCTAGTCTCCAGGCACGCATCACACCTATACATCCGAT

Supplemental Table 7. 601 DNA used for nucleosome binding assay

Modified 601 DNAs used in nucleosome reconstitution (Fig. 4) are listed. To design these DNAs, we used the following structural information and DNA substrates: [6]; [15].

References

1. UniProt Consortium: **UniProt: a hub for protein information.** *Nucleic Acids Res* 2015, **43:**D204-212.
2. Menafra R, Brinkman AB, Matarese F, Franci G, Bartels SJ, Nguyen L, Shimbo T, Wade PA, Hubner NC, Stunnenberg HG: **Genome-wide binding of MBD2 reveals strong preference for highly methylated loci.** *PLoS ONE* 2014, **9:**e99603.
3. Adomas AB, Grimm SA, Malone C, Takaku M, Sims JK, Wade PA: **Breast tumor specific mutation in GATA3 affects physiological mechanisms regulating transcription factor turnover.** *BMC Cancer* 2014, **14:**278.
4. Taguchi H, Horikoshi N, Arimura Y, Kurumizaka H: **A method for evaluating nucleosome stability with a protein-binding fluorescent dye.** *Methods* 2014, **70:**119-126.
5. Chen Y, Bates DL, Dey R, Chen PH, Machado AC, Laird-Offringa IA, Rohs R, Chen L: **DNA binding by GATA transcription factor suggests mechanisms of DNA looping and long-range gene regulation.** *Cell Rep* 2012, **2:**1197-1206.
6. Vasudevan D, Chua EY, Davey CA: **Crystal structures of nucleosome core particles containing the '601' strong positioning sequence.** *J Mol Biol* 2010, **403:**1-10.
7. Emsley P, Cowtan K: **Coot: model-building tools for molecular graphics.** *Acta Crystallogr D Biol Crystallogr* 2004, **60:**2126-2132.
8. Kim D, Pertea G, Trapnell C, Pimentel H, Kelley R, Salzberg SL: **TopHat2: accurate alignment of transcriptomes in the presence of insertions, deletions and gene fusions.** *Genome Biol* 2013, **14:**R36.
9. Anders S, Pyl PT, Huber W: **HTSeq--a Python framework to work with high-throughput sequencing data.** *Bioinformatics* 2015, **31:**166-169.
10. Love MI, Huber W, Anders S: **Moderated estimation of fold change and dispersion for RNA-seq data with DESeq2.** *Genome Biol* 2014, **15:**550.
11. Trapnell C, Roberts A, Goff L, Pertea G, Kim D, Kelley DR, Pimentel H, Salzberg SL, Rinn JL, Pachter L: **Differential gene and transcript expression analysis of RNA-seq experiments with TopHat and Cufflinks.** *Nat Protoc* 2012, **7:**562-578.
12. Buenrostro JD, Giresi PG, Zaba LC, Chang HY, Greenleaf WJ: **Transposition of native chromatin for fast and sensitive epigenomic profiling of open chromatin, DNA-binding proteins and nucleosome position.** *Nat Methods* 2013, **10:**1213-1218.
13. Langmead B, Trapnell C, Pop M, Salzberg SL: **Ultrafast and memory-efficient alignment of short DNA sequences to the human genome.** *Genome Biol* 2009, **10:**R25.
14. Cui K, Zhao K: **Genome-wide approaches to determining nucleosome occupancy in metazoans using MNase-Seq.** *Methods Mol Biol* 2012, **833:**413-419.
15. Makde RD, England JR, Yennawar HP, Tan S: **Structure of RCC1 chromatin factor bound to the nucleosome core particle.** *Nature* 2010, **467:**562-566.

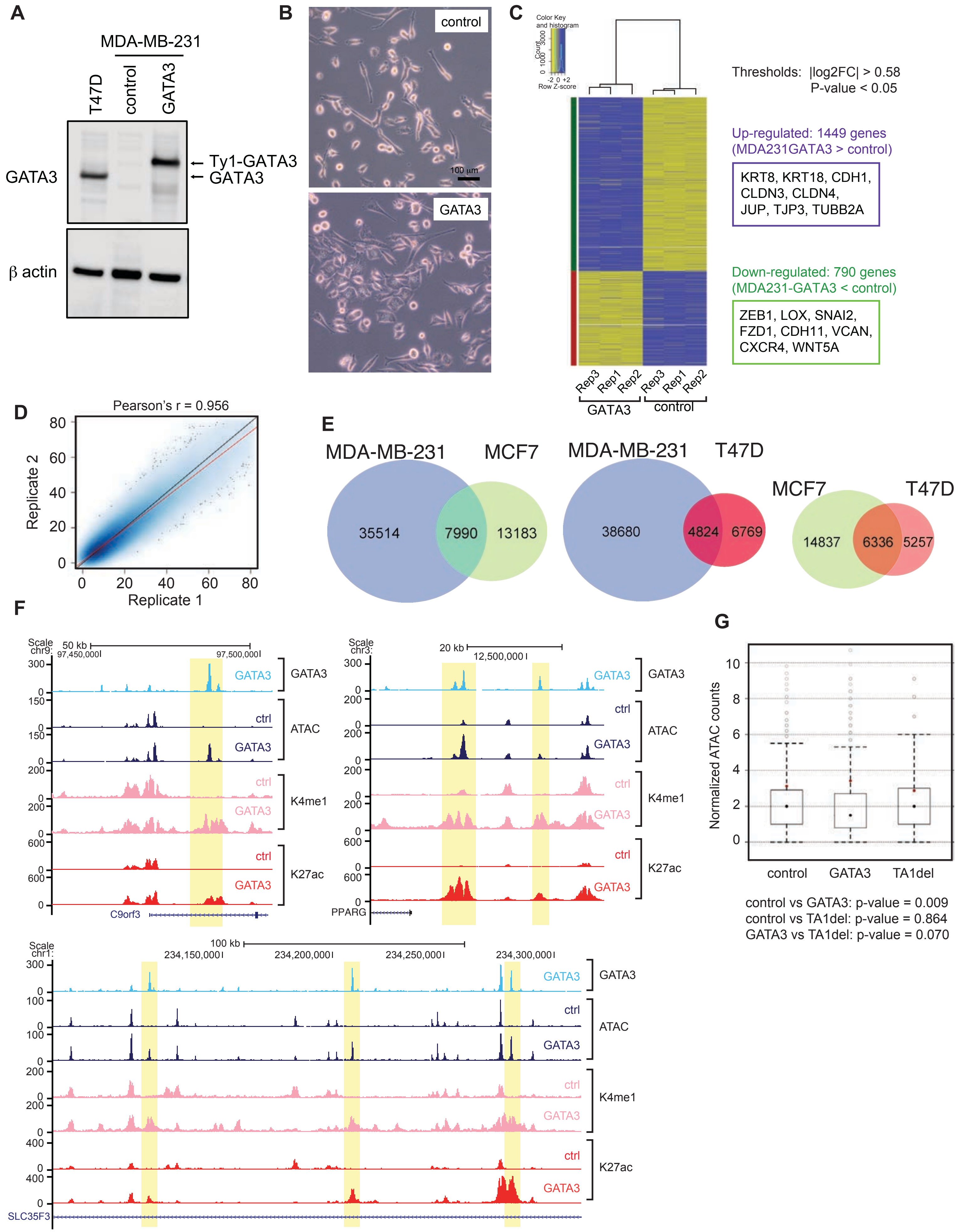
A
MDA-MB-231
T47D
control
GATA3
GATA3
← Ty1-GATA3
← GATA3
β actin
B
control
100 μm
GATA3
C
Color Key and histogram
Count
Row Z-score
Thresholds: |log2FC| > 0.58
P-value < 0.05
Up-regulated: 1449 genes
(MDA231GATA3 > control)
KRT8, KRT18, CDH1, CLDN3, CLDN4, JUP, TJP3, TUBB2A
Down-regulated: 790 genes
(MDA231-GATA3 < control)
ZEB1, LOX, SNAI2, FZD1, CDH11, VCAN, CXCR4, WNT5A
Rep3 Rep1 Rep2 Rep3 Rep1 Rep2
GATA3
control
D
Pearson's r = 0.956
Replicate 2
Replicate 1
E
MDA-MB-231
MCF7
35514
7990
13183
MDA-MB-231
T47D
38680
4824
6769
MCF7
T47D
14837
6336
5257
F
GATA3
ATAC
ctrl
K4me1
K27ac
C9orf3
PPARG
SLC35F3
G
Normalized ATAC counts
control
GATA3
TA1del
control vs GATA3: p-value = 0.009
control vs TA1del: p-value = 0.864
GATA3 vs TA1del: p-value = 0.070

Takaku_Supplemental Fig2

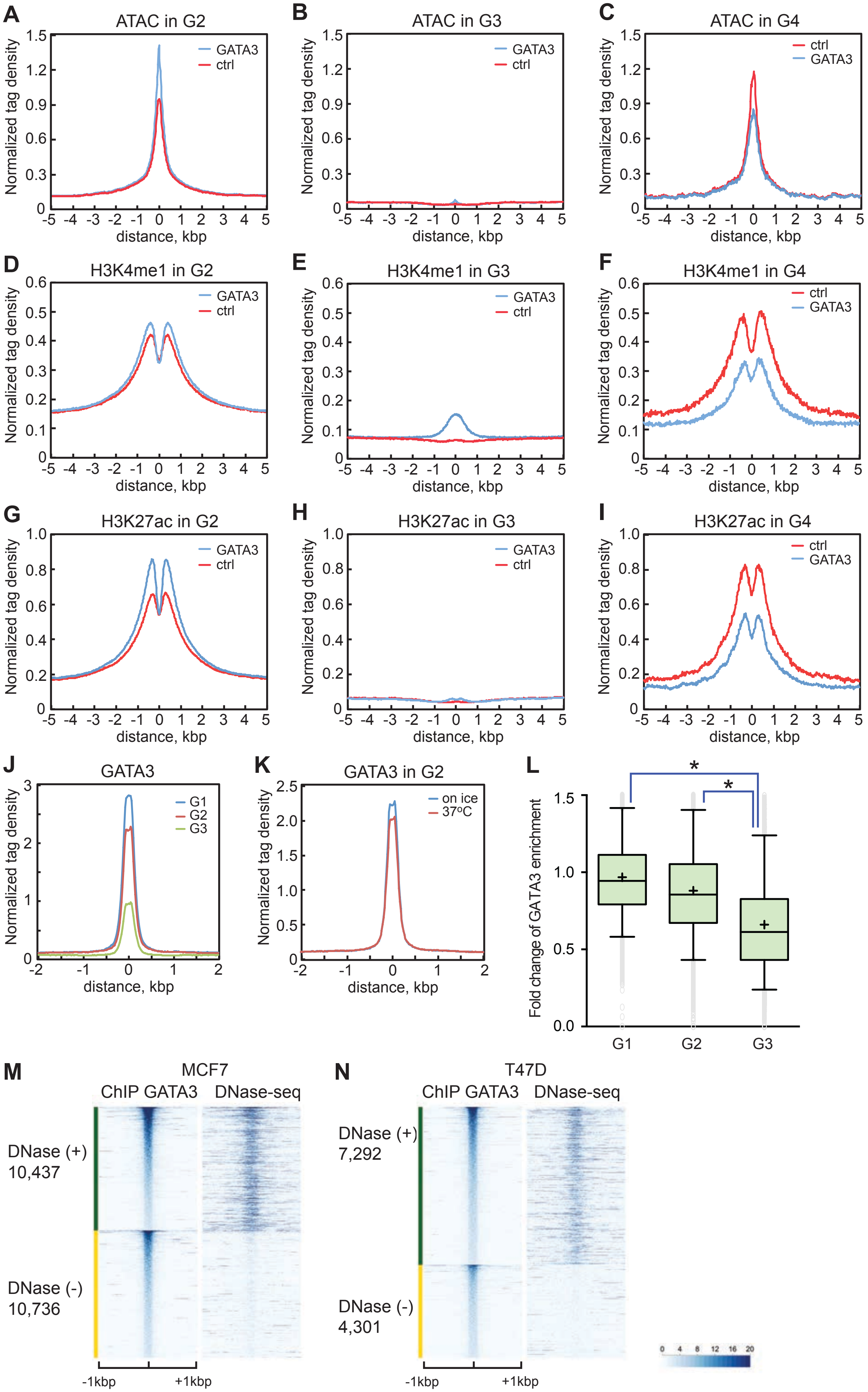

Takaku et al_Figure S3

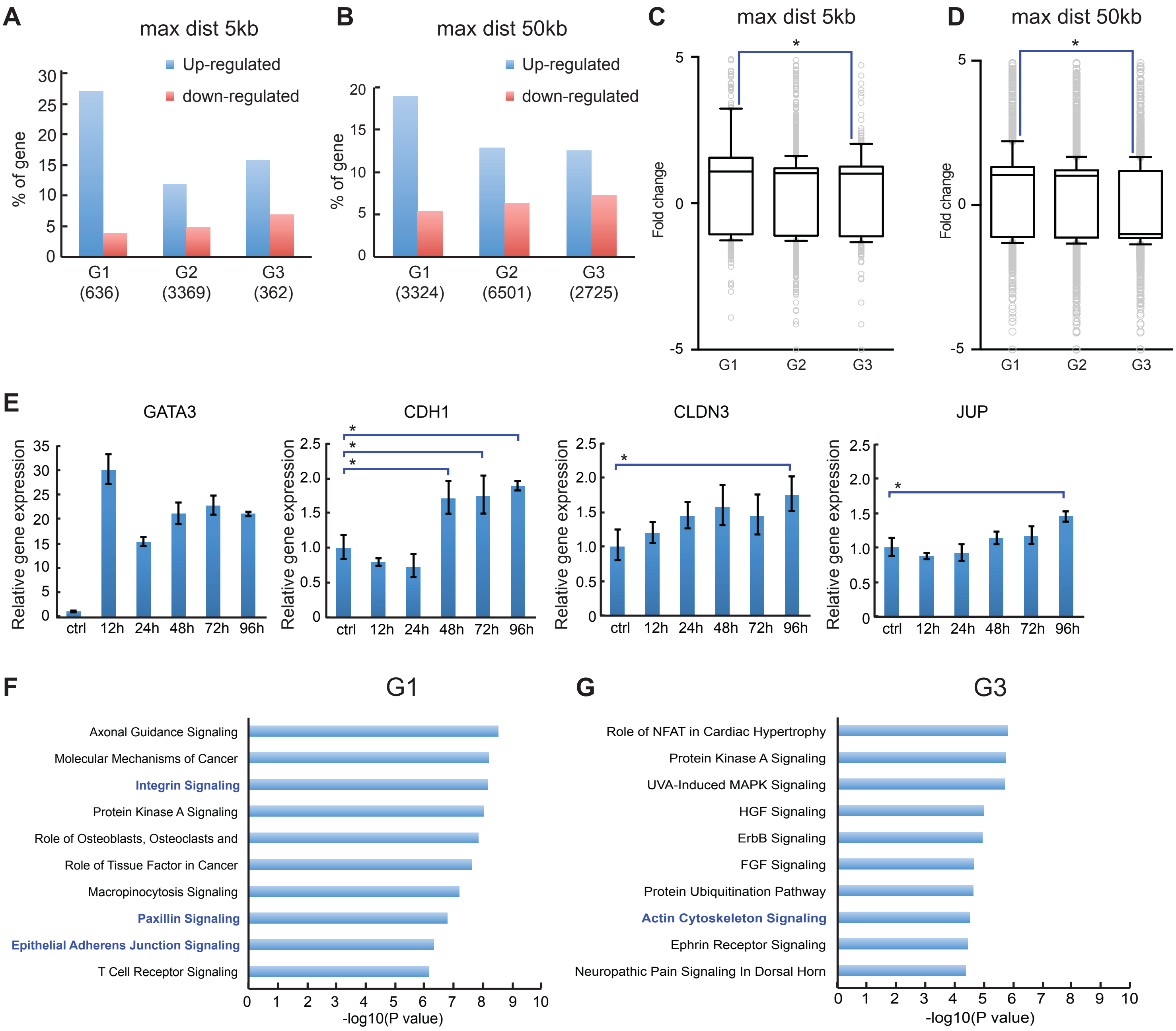

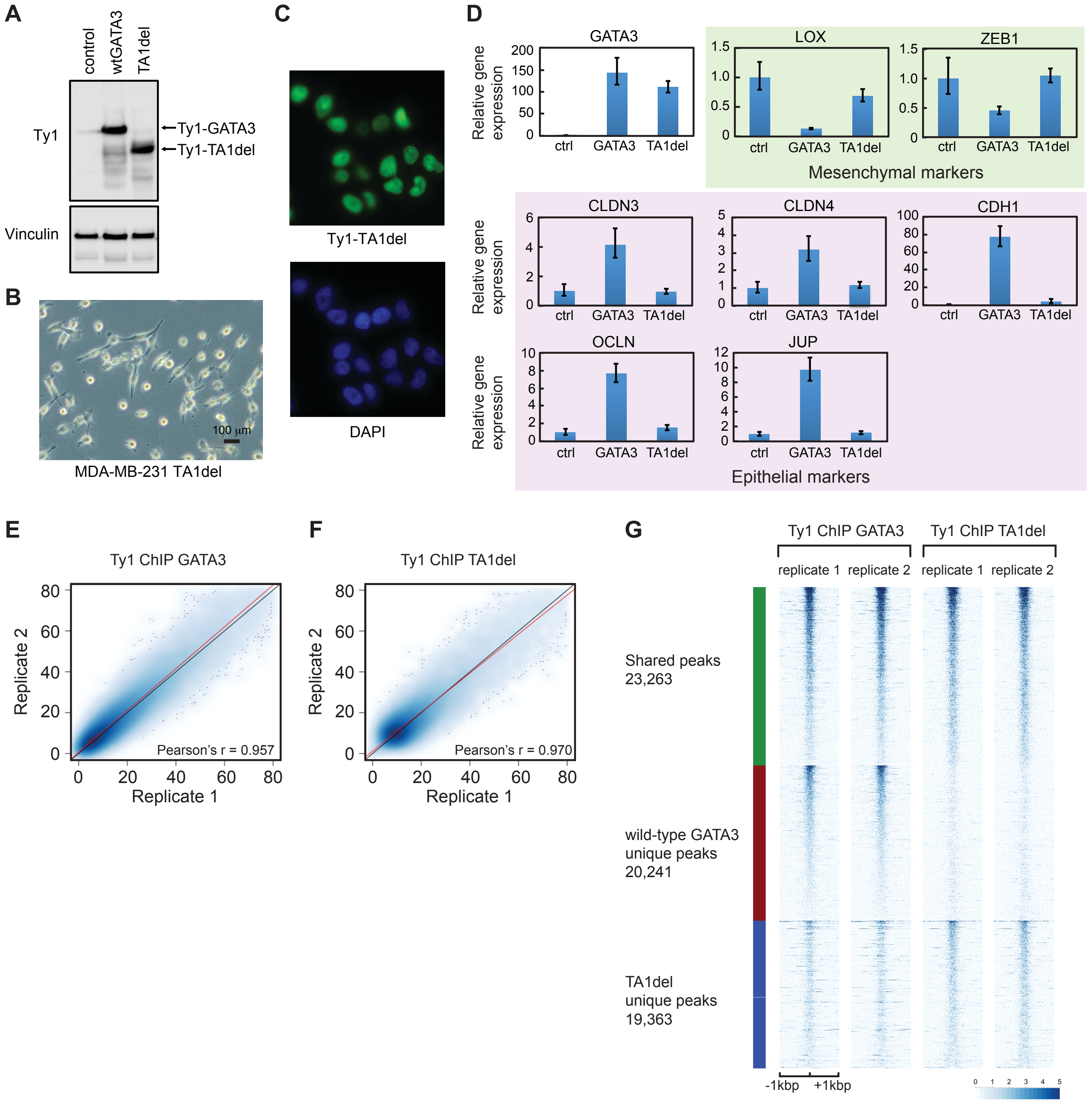
A
control
wtGATA3
TA1del
Ty1
←Ty1-GATA3
←Ty1-TA1del
Vinculin
B
100 μm
MDA-MB-231 TA1del
C
Ty1-TA1del
DAPI
D
Relative gene expression
GATA3
LOX
ZEB1
ctrl
GATA3
TA1del
Mesenchymal markers
CLDN3
CLDN4
CDH1
OCLN
JUP
Epithelial markers
E
Ty1 ChIP GATA3
Replicate 2
Replicate 1
Pearson's r = 0.957
F
Ty1 ChIP TA1del
Pearson's r = 0.970
G
Ty1 ChIP GATA3
Ty1 ChIP TA1del
replicate 1
replicate 2
Shared peaks
23,263
wild-type GATA3
unique peaks
20,241
TA1del
unique peaks
19,363
-1kbp
+1kbp

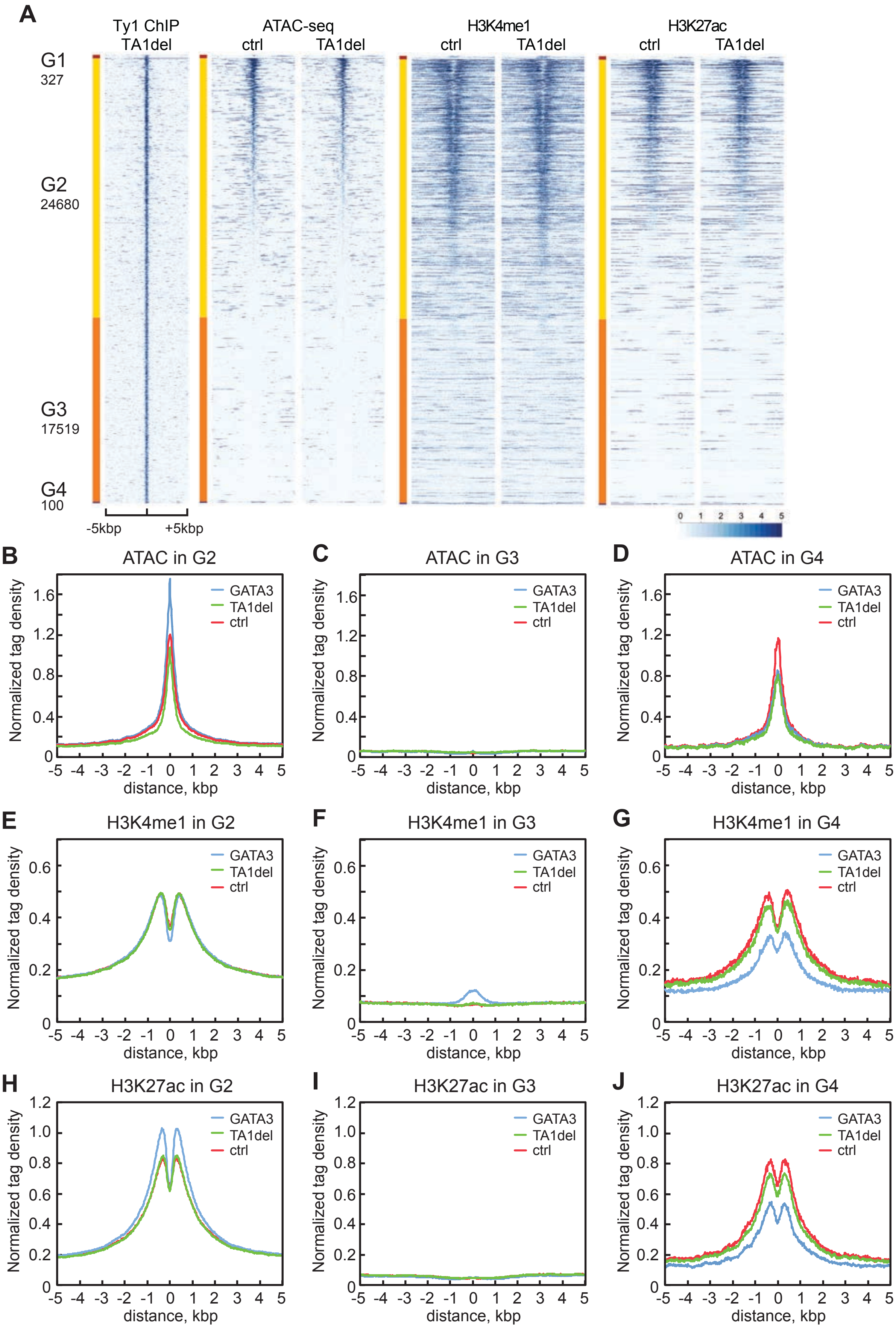
A
Ty1 ChIP
TA1del
ATAC-seq
ctrl
TA1del
H3K4me1
ctrl
TA1del
H3K27ac
ctrl
TA1del
G1
327
G2
24680
G3
17519
G4
100
-5kbp
+5kbp
0 1 2 3 4 5
B
ATAC in G2
C
ATAC in G3
D
ATAC in G4
E
H3K4me1 in G2
F
H3K4me1 in G3
G
H3K4me1 in G4
H
H3K27ac in G2
I
H3K27ac in G3
J
H3K27ac in G4
GATA3
TA1del
ctrl
Normalized tag density
distance, kbp

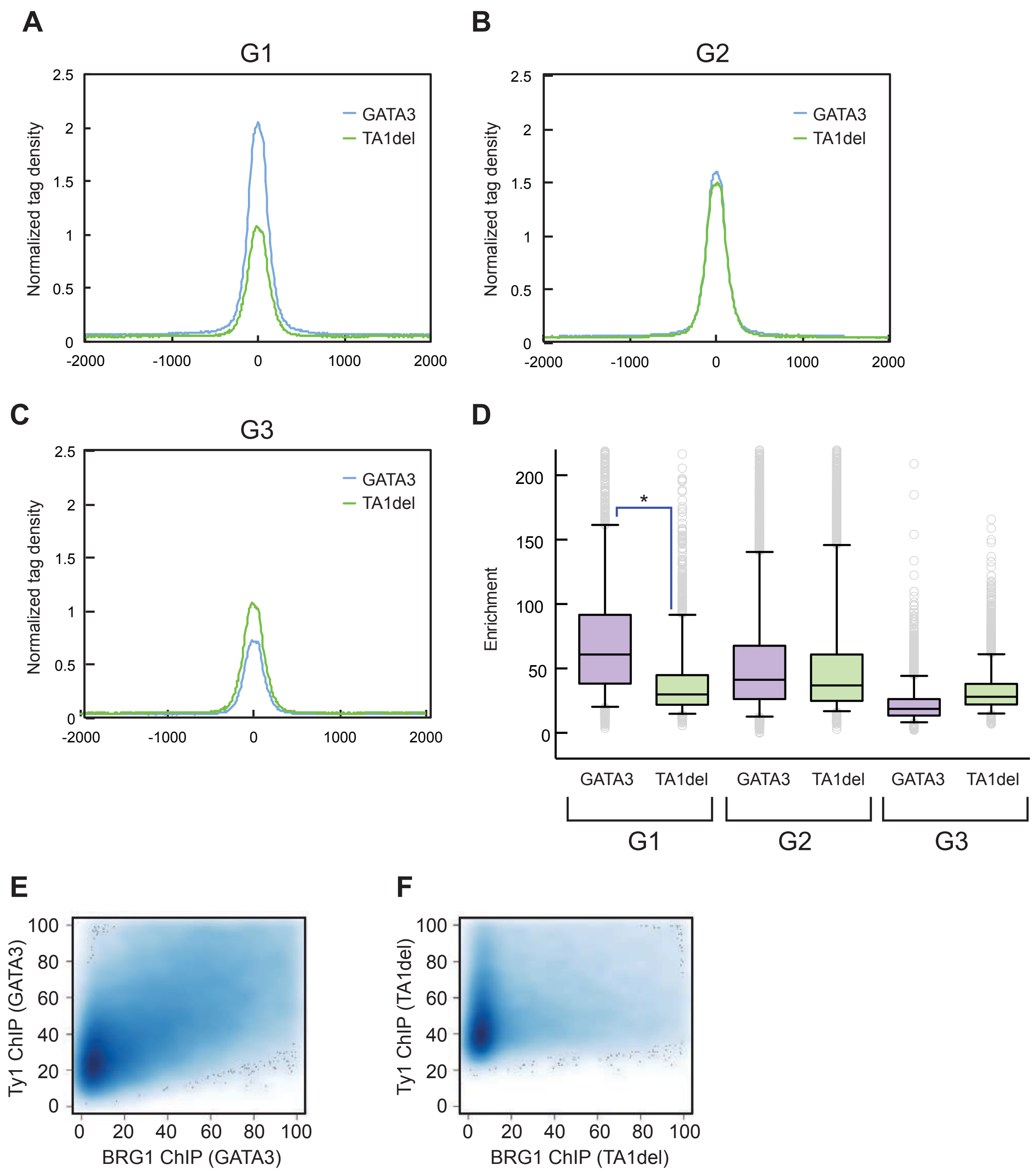
A
G1
Normalized tag density
GATA3
TA1del
B
G2
C
G3
D
Enrichment
*
GATA3
TA1del
G1
G2
G3
E
Ty1 ChIP (GATA3)
BRG1 ChIP (GATA3)
F
Ty1 ChIP (TA1del)
BRG1 ChIP (TA1del)
